# Gait classification for growing children with Duchenne muscular dystrophy

**DOI:** 10.1038/s41598-024-61231-y

**Published:** 2024-05-11

**Authors:** Ines Vandekerckhove, Eirini Papageorgiou, Britta Hanssen, Nathalie De Beukelaer, Marleen Van den Hauwe, Nathalie Goemans, Anja Van Campenhout, Liesbeth De Waele, Friedl De Groote, Kaat Desloovere

**Affiliations:** 1 Department of Rehabilitation Sciences, KU Leuven, Leuven, Belgium; 2 Department of Surgery, University of Geneva, Geneva, Switzerland; 3 Department of Child Neurology, University Hospital Leuven, Leuven, Belgium; 4 Department of Development and Regeneration, KU Leuven, Leuven, Belgium; 5 Department of Orthopedics, University Hospital Leuven, Leuven, Belgium; 6 Department of Movement Sciences, KU Leuven, Leuven, Belgium; 7 Clinical Motion Analysis Laboratory, University Hospital Leuven, Pellenberg, Belgium

**Keywords:** Neuromuscular disease, Orthopaedics, Paediatric research

## Abstract

Classifying gait patterns into homogeneous groups could enhance communication among healthcare providers, clinical decision making and clinical trial designs in boys with Duchenne muscular dystrophy (DMD). Sutherland’s classification has been developed 40 years ago. Ever since, the state-of-the-art medical care has improved and boys with DMD are now longer ambulatory. Therefore, the gait classification requires an update. The overall aim was to develop an up-to-date, valid DMD gait classification. A total of 137 three-dimensional gait analysis sessions were collected in 30 boys with DMD, aged 4.6–17 years. Three classes were distinguished, which only partly aligned with increasing severity of gait deviations. Apart from the mildly affected pattern, two more severely affected gait patterns were found, namely the tiptoeing pattern and the flexion pattern with distinct anterior pelvic tilt and posterior trunk leaning, which showed most severe deviations at the ankle or at the proximal segments/joints, respectively. The agreement between Sutherland’s and the current classification was low, suggesting that gait pathology with the current state-of-the-art medical care has changed. However, overlap between classes, especially between the two more affected classes, highlights the complexity of the continuous gait changes. Therefore, caution is required when classifying individual boys with DMD into classes.

## Introduction

Duchenne muscular dystrophy (DMD) is a severe X-linked degenerative disorder, affecting one in 3500–6000 newborn boys^1–3^. DMD is caused by mutations in the dystrophin gene and subsequent dystrophin deficiency, which results in progressive muscle degeneration characterized by loss of contractile tissue and replacement by fat and fibrotic tissue^2,4^. Clinical symptoms of DMD include progressive muscle weakness, muscle stiffness, contractures, delayed motor milestones and a decline in motor function, eventually resulting in loss of ambulation^1,2,5,6^. If untreated, boys with DMD become wheelchair-bound around 9 years of age and die around 19 years due to pulmonary infections or cardiac failure^5,7^. To date, multidisciplinary symptomatic medical, orthopedic, and rehabilitative treatment, including long-term use of corticosteroids, and ventilatory support, have led to delayed loss of walking ability, increased life expectancy and improved quality of life for boys with DMD^2,5,8–10^. Prolonging ambulation is considered an important treatment goal in order to maintain a certain level of functionality and to postpone the development of contractures and bony deformities in children with DMD^1,2^.

Promising novel therapeutic strategies may further slow disease progression and delay loss of ambulation, but clinical development of novel therapies has been hampered by the challenges to design and perform clinical trials^11–14^. Both the rarity and variability of DMD have complicated clinical trial design, highlighting the importance to homogenize the included study sample. Additionally, sensitive outcome measures, that capture subtle changes induced by disease progression and/or treatment, are lacking^11–14^. Currently, functional tests, such as the 6-min walk test (6MWT), the North Star Ambulatory Assessment (NSAA) and timed tests, are frequently used as evaluation tools in clinical trials^14–16^. Although these tests are found to be reliable, valid and feasible^17–20^, the high variability in test outcomes has confounded the accurate detection of treatment effects, despite the efforts to homogenize the included study sample^13^. Recently, the European Medicines Agency qualified stride velocity 95th centile, which is a measure of peak performance and represents the speed of the fastest strides recorded over a period of 180 hours, for use as secondary endpoint in pivotal studies for DMD^21,22^. Yet, clinical endpoints on the quality of gait are lacking. Furthermore, comprehensive knowledge of the disease progression in boys with DMD, who are monitored in routine clinical practice, plays a significant role in defining and characterizing the effects of novel therapies. Therefore, ameliorated trial designs, sensitive and clinical meaningful outcome measures, and natural history data are required to overcome the difficulties in clinical development^11,14^.

Evaluating gait quality by means of three-dimensional gait analysis (3DGA) has been proposed to monitor the disease progression in clinical practice as well as to prove the efficacy of novel therapeutic strategies in clinical trials^23–26^. Romano et al.^24^ indicated the added value of 3DGA to global function tests, since the strength of the correlations between 3DGA parameters, and 6MWT or NSAA varied considerably. Additionally, improved hip kinetics were observed after the start of corticosteroids^23^ and a slower rate of decline in gait quality was detected in boys on corticosteroids compared to steroid-naïve boys^27^. This highlights the potential of 3DGA to assist in the detection of treatment efficacy in clinical trials.

The use of 3DGA in clinical trials necessitates a full delineation of the DMD gait pathology. DMD gait has been characterized by reduced walking velocity, smaller step length, larger anterior pelvic tilt, reduced hip extension, increased knee range of motion, decreased dorsiflexion in stance and swing, smaller knee extension and dorsiflexion moments, and diminished hip and ankle power generation, compared to typically developing (TD) children^16,24,26^. However, mostly cross-sectional studies including a small number of participants with a wide age range have been performed^16^. Since most previous studies only reported the averages of children that are located at different stages of the disease progression, knowledge of the full spectrum of the progressive gait pathology was lacking^26^. Through a longitudinal 5-year follow-up study, we recently described the evolution in gait features in growing boys with DMD^26^. Especially the progressive gait pathology towards more anterior pelvic tilt, hip flexion, internal foot progression and less dorsiflexion at initial contact was confirmed^26^. A later longitudinal study by Sienko et al.^27^ confirmed these results as the gait variable scores of sagittal pelvis, hip and ankle kinematics, coronal pelvis and hip kinematics, and transversal foot progression angle revealed significant rates of increasing deviations with age. A decline in overall quality of gait was also evident in growing boys with DMD^26,27^. Yet, high variability in progression rate, as well as age-dependency among individual patients, pose a challenge to easily interpret the longitudinal delineation of DMD gait^26^.

Classifying gait patterns into homogeneous groups could facilitate descriptions of gait and therefore enhance communication among healthcare providers, clinical decision making and clinical trial designs. In 1981, Sutherland et al.^1^ developed the first DMD gait classification. Based on cadence, maximal anterior pelvic tilt in stance, and maximal dorsiflexion during swing, children with DMD were classified into the early, transitional or late stage with an accuracy of 91%^1^. However, the stages of Sutherland have been introduced more than 40 years ago^1^. Due to improved state-of-the-art medical care with long-term use of corticosteroids, children with DMD are now ambulant for a longer period of time, approximately 2.1–4.4 years^28,29^. This altered treatment management may have also influenced the gait patterns of boys with DMD. A second gait classification from Sienko Thomas et al.^30^ classified children with DMD based on the gait deviation index (GDI) into a mild (mean ± standard deviation of GDI: 84.96 ± 14), moderate (74.80 ± 13) or advanced group (69.57 ± 14). Although similarities were found between the groups of Sienko Thomas et al.^30^ and the stages of Sutherland et al.^1^, the two classifications were not completely comparable. Therefore, an updated DMD gait classification is currently lacking.

The overall aim was to develop an up-to-date, valid DMD gait classification, covering the entire ambulation period, by applying machine-learning techniques based on an extensive longitudinal gait database. The first aim was to identify groups of similar patterns within the gait pathology of children with DMD following a data-driven approach. This aim was investigated from a cross-sectional perspective, because we focused on the detection of gait patterns present in children with DMD, irrespectively of the highly variable individual progression rates of gait pathology. This first aim was further divided into detecting the optimal number of data-driven gait patterns (“Aim 1.a”) and checking the robustness of the classification by performing a k-fold cross-validation (“Aim 1.b”). We hypothesized that the optimal number of gait patterns following a data-driven approach would be more than three, due to prolonged ambulation since the first gait classification in DMD (“Hypothesis Aim 1.a”). Additionally, we hypothesized that the misclassification rate would be less than 10%, reflecting a robust classification (“Hypothesis Aim 1.b”). The second aim was to describe and compare the characteristics of the data-driven gait patterns to the gait pattern of TD children (“Aim 2.a”) and among the data-driven gait patterns (“Aim 2.b”). We hypothesized to find a mildly, a moderately and a severely affected gait pattern based on the previously reported stages by Sutherland et al.^1^ and Sienko Thomas et al.^30^ (“Hypothesis Aim 2”). The third aim was to investigate whether the progressive gait pathology of boys with DMD who received the current state-of-the-art medical care has changed compared to the natural history study of Sutherland et al.^1^, where boys had not received any medical interventions. Based on preliminary results and the fact that Sutherland et al. is more than 40 years old^1^, we hypothesized that the current progressive gait pathology has changed (“Hypothesis Aim 3”).

## Methods

### Patients

Thirty-four boys with DMD were recruited at the Neuromuscular Reference Centre in the University Hospital Leuven campus Gasthuisberg. The patients were included based on the following criteria: between 3 and 16 years old at baseline (1), confirmed genetic diagnosis of DMD (2), and ability to walk independently for at least 100 meters (3). Long-term use of corticosteroids and clinical trial participation with disease-modifying medication were allowed. Cognitive and behavioral disorders preventing accurate measurements (1), clinical picture of Becker muscular dystrophy (2), and history of muscle lengthening surgery (3), were the exclusion criteria. From the reference database from the Clinical Motion Analysis Laboratory in the University Hospital Leuven campus Pellenberg (CMAL-Pellenberg), 19 TD boys, who had no history of neurological or neuromuscular problems and who had a similar age-range as the boys with DMD, were selected.

The local ethics committee (Ethical Committee UZ Leuven/KU Leuven; S55867, S56041, and S61324) approved this study under the Declaration of Helsinki. All methods were performed in accordance with the relevant guidelines and regulations. Prior to participation, a written informed consent was signed by participants’ parents/caregivers. Participants of 12 years or older signed an assent.

### Data collection

Five subject characteristics were collected, including age (years), leg length (i.e., distance between the lower edge of the anterior superior iliac spine and the lower border of the medial malleolus, in m), body mass index (BMI; in kg/m^2^), body mass (in kg), and body height (in m). Gait was measured by 3DGA’s. The children walked barefoot on a 10-m walkway at self-selected walking speed. The Plug-In Gait Full-Body and the Plug-In Gait Lower-Body reflective marker (diameter of 14 mm) models were attached to the skin of the boys with DMD and TD boys, respectively. The marker trajectories were captured by a 10–15 Vicon camera system (Vicon-UK, Oxford, United Kingdom) with a sampling frequency of 100 Hz and were filtered with a built-in Woltring filter (mode: MSE; smoothing: 15 mm^2^). Ground reaction forces were registered at 1500 Hz by two force plates (AMTI, Watertown, MA, United States) embedded in the walkway. Internal joint moments were calculated by combining the marker trajectories with the ground reaction forces. A digital video recording system was synchronized with the Vicon system to provide qualitative data of the walking motion. In the Nexus software (Nexus 2.10, Vicon-UK, Oxford, United Kingdom), gait cycles (GCs) were defined by manually indicating the gait events, with the support of force plate data if available, and 12 joint kinematic waveforms (i.e., sagittal, coronal and transversal plane kinematics of trunk, pelvis, hip, knee and ankle segments/joints) as well as seven kinetic waveforms (i.e., sagittal plane moment and power of hip, knee and ankle joints, and coronal plane moment of hip joint) were estimated (Table S1). Three to five GCs with kinematic and kinetic data were collected for the left and right side. The number of collected GCs depended on the child’s level of fatigue and cooperation.

### Data analysis

A custom-made software in MATLAB (The Mathworks Inc., Natick, M.A., 2016 and 2021b) was used to evaluate the quality of the collected GCs (more details below). Four spatiotemporal parameters were calculated, including cadence (steps/s), step length, step width and walking velocity. Step length and step width were normalized to leg length (Eqs. 1 and 2), while walking velocity was normalized to leg length and gravitational acceleration (Eq. 3), according to the equations of Hof ^31^ (Table S1):

1 $$Normalized \,\,step \,\,length=\frac{Step \,\,length}{leg \,\,length}$$

2 $$Normalized \,\,step \,\,width=\frac{Step \,\,width}{leg \,\,length}$$

3 $$Normalized \,\,walking \,\,velocity=\frac{Walking \,\,velocity}{\sqrt{leg \,\,length*gravitational \,\,acceleration}}$$

Nineteen kinematic and kinetic waveforms were resampled to 101 data points per GC (Table S1). Kinematic waveforms were expressed in degrees, internal joint moments in Newton meters per kilogram body mass (Nm/kg), and power waveforms in Watt per kilogram body mass (W/kg).

The collected GCs were classified into the early, transitional or late stage of Sutherland’s classification using their Fisher Linear Discriminant formula (Table S2)^1^. This formula is based on one spatiotemporal parameter, i.e., cadence expressed in steps per minute, and on two discrete parameters, i.e., maximal anterior pelvic tilt angle in stance and maximal ankle dorsiflexion in swing, which were calculated from the respective kinematic waveforms (Table S1).

### Dataset

The boys with DMD were repeatedly measured between 2015 and 2022 at multiple time points (one to ten) with a variable time interval of 5–35 months, covering a follow-up period of 6 months–6 years. In total, the collected dataset consisted of 152 3DGA sessions of 34 boys with DMD. The flowchart in Fig. S1 summarizes the data handling. After the quality check, 15 3DGA sessions of 12 patients (including all sessions of four boys) were excluded due to obvious marker misplacement or detachment, inconsistent walking and/or behavioral disturbances leading to inaccurate measurements, resulting in a dataset of 137 3DGA sessions of 30 boys with DMD, aged 4.6–17 years. An overview of all the included 3DGA sessions per age and per boy with DMD is provided in Fig. S2. Subsequently, we checked whether the dataset was imbalanced, i.e., whether we expected an unequal distribution across stages of the disease progression. This required a rough preliminary classification based on the two previous classifications^1,30^ and insights from the clinical experts at CMAL-Pellenberg. According to this rough estimation, 77, 42 and 18 3DGA sessions were classified into the mildly, moderately and severely affected gait patterns, respectively, confirming an imbalanced dataset with an overrepresentation of less-affected children (Fig. S1). To solve this imbalance, we applied the technique of combining undersampling of the majority classes with oversampling of the minority class, which has been widely considered in literature as data preprocessing step for imbalanced datasets^32–37^. In specific, all 3DGA sessions of the mildly and moderately affected groups were included, but these classes were undersampled by randomly selecting one GC per side for the mildly affected group and one to two GCs per side for the moderately affected group per 3DGA session (Fig. S1). The severely affected group was oversampled, not by duplicating or synthetic methods^34,35^, but by including all available GCs per 3DGA session (Fig. S1). This resulted in a more balanced dataset, i.e., the mildly affected group consisted of 154 included GCs, the moderately affected group of 136 GCs and the severely affected group of 140 GCs. In total, 430 GCs were included in further analyses (Fig. S1). The sole purpose of the rough preliminary classification was to solve the imbalanced dataset. After resampling, this classification was no longer taken into account.

### Statistical analysis

#### Aim 1: to identify groups of similar patterns within the gait pathology, by detecting the optimal number of data-driven gait patterns (Aim 1.a) and by checking the robustness of the classification (Aim 1.b)

To detect the optimal number of gait patterns following a data-driven approach (“Aim 1.a”), we first performed principal component analyses (PCA) and then a k-means cluster analysis in R (version 4.2.0, R Foundation for Statistical Computing, Vienna, Austria).

The waveform of the hip internal rotation angle and the waveform of the hip abduction moment were excluded from the analyses to detect gait patterns, because hip internal rotation angle is prone to error^38^ and the maximal hip abduction moment presented a less clear longitudinal evolution^26^. All 17 remaining waveforms were included (Table S3).

Before running the PCAs, data was standardized (centered and scaled). For each of the 17 continuous waveforms, a separate PCA was performed (430 GCs × 101 data points, for 17 waveforms (Table S3)). One PCA was performed on the spatiotemporal parameters (430 GCs × 4 parameters (i.e., cadence, normalized step length, normalized step width, normalized walking velocity)). Through PCA, the multivariate dataset was reduced into principal components (PCs) (i.e., uncorrelated linear combinations of the original parameters), which were ordered by the amount of explained variability. The PCs that expressed a cumulative variability of 80% were retained for the spatiotemporal parameters and for each waveform. Thereafter, the PC scores were calculated for each retained PC.

A k-means cluster analysis was conducted on the PC scores of the retained PCs to detect homogeneous patterns. A consensus-based algorithm was applied to determine the optimal number of clusters (‘n_clusters()’ function of parameters package available in R)^39^. Hereby, 30 different methods (e.g., Gap statistics or Silhouette method) were calculated for one to ten clusters (ten iterations). The final optimal number of clusters was determined by the number of clusters on which the majority of the methods agreed (i.e., find the consensus). After performing the k-means cluster analysis with the optimal number of clusters, the distance of each GC to each cluster center was calculated to assign the membership of GCs to the closest cluster.

To determine the robustness of the classification (“Aim 1.b”), a 30-fold cross-validation was performed. Therefore, the membership of the GCs based on the k-means cluster analysis using all data served as the *reference* membership, and the dataset was split into folds per participant (i.e., one fold consisted of all the GCs of all the measurements of one boy). Then, the PCAs and k-means cluster analysis were repeated leaving one fold out. For the GCs of the held out fold, the PC scores were predicted and used to determine a *test* membership to the new clusters derived from the dataset in which the fold was left out. This process was repeated for each fold, i.e., 30 times. This way, each GC had a reference and test membership. Subsequently, a confusion matrix, sensitivity and specificity per cluster and overall misclassification rate were calculated^40^.

#### Aim 2: to describe and compare the characteristics of the data-driven gait patterns to the gait pattern of TD children (Aim 2.a) and among the data-driven gait patterns (Aim 2.b)

Normality of the subject characteristics and spatiotemporal parameters was checked by inspecting the distribution plots and conducting Shapiro–Wilk tests. To compare kinematic and kinetic waveforms, statistical (non-)parametric mapping (SPM-SPM1d version 0.4.7, http://www.spm1d.org/) was used^41^, with 10,000 iterations. Normality of this data was assessed with a built-in function in S(n)PM. Since most of the parameters were not normally distributed, non-parametric statistics were performed. All statistical analyses concerning this aim were conducted in MATLAB (2021b).

##### Aim 2.a

Differences in spatiotemporal parameters between TD gait and each DMD gait pattern were examined with Mann–Whitney U tests. According to the Bonferroni correction^42^, the significance threshold was set to α = 0.004 to correct for the four spatiotemporal parameters (i.e., cadence, normalized step length, normalized step width, normalized walking velocity) in three pairwise comparisons (i.e., cluster one vs TD, cluster two vs TD, cluster three vs TD). To compare the gait waveforms of each DMD gait pattern to the gait pattern of the TD group, two sample Hotelling’s T^2^ tests were first performed on a vector level (i.e., combination of waveforms per motion plane: sagittal, coronal, transversal kinematics, sagittal moments, powers)^43^. Hereto, the significance threshold was set to α = 0.017, correcting for three pairwise comparisons. If significant, subsequent post-hoc, two sample t-tests were applied on segment/joint level (i.e., pelvis, hip, knee and ankle) with the significance threshold ranging from 0.004 to 0.017, to consider the number of segments/joints per motion plane of the kinematics, moments and powers in three pairwise comparisons (Table S4).

##### Aim 2.b

Kruskal–Wallis tests were performed to determine whether the continuous variables, namely five subject characteristics (age, leg length, BMI, body mass and body height) and four spatiotemporal parameters (cadence, normalized step length, normalized step width, normalized walking velocity), were different among gait patterns. The significance threshold was set to α = 0.006, to correct for the comparison of these nine parameters. In case of significance, post-hoc Mann–Whitney U tests were conducted to locate these differences. The significance threshold was set to α = 0.002 to correct for the nine parameters in three pairwise comparisons (i.e., cluster one vs cluster two, cluster two vs cluster three, cluster three vs cluster one). For the gait waveforms, MANOVA (multivariate analysis of variance) tests (α = 0.05) were applied to compare vectors among the gait patterns, with post-hoc two sample Hotelling’s T^2^ tests to locate the differences between gait patterns on a vector level (Bonferroni corrected α = 0.017) and subsequent post-hoc two sample t-tests on segment/joint level (Bonferroni corrected α ranging from 0.003 to 0.017 (Table S5)).

After determining statistical significance, the clinical relevance of these findings was judged. Following an approach that was previously established^44^, the differences in spatiotemporal parameters should exceed previously reported minimal detectable changes (MDC)^45^. Per suprathreshold cluster from the SPM results, the duration should be longer or equal to 3% of the GC and the differences in the segment/joint waveforms between groups should be larger than the respective standard errors of measurements (SEM) for the intra-rater intersession reliability in TD by Kainz et al.^38^ (for pelvis and lower limb) and Wilken et al.^46^ (for trunk) for 80% or more of the suprathreshold cluster’s duration. Due to the lack of the SEM for the powers and the foot progression angle, the changes in these waveforms could not be clinically judged^38^.

#### Aim 3: to compare the current classification with Sutherland’s classification

To compare the current classification with Sutherland’s classification, a contingency table was generated between the cluster reference memberships and the assignments to Sutherland’s classification. Percentages were used to indicate the relative proportion of the GCs classified as Sutherland’s early, transitional and late stage per cluster. A total percentage of agreement was also calculated by first summing the number of GCs, where the early, transitional, and late stage assignment corresponded with cluster one, two, and three membership, respectively, and then dividing this number by the total number of GCs and multiplying it by 100.

## Results

### Aim 1: to identify groups of similar patterns within the gait pathology

Two and 68 PCs were retained for the spatiotemporal parameters and waveforms, respectively (Table S3). Ten out of 30 methods indicated three as the optimal number of clusters (Fig. S3). The between sum of squares to total sum of squares ratio was only 23.7%, indicating that the clusters were not well separated and compact. Based on the three-cluster configuration, 181 GCs were assigned to cluster one, 59 GCs to cluster two and 190 GCs to cluster three. The classification showed an overall high robustness by the 30-fold cross-validation, with a misclassification rate of 8.6% (Table 1). The sensitivity and specificity for the separate clusters ranged between 87.5–99.7%, with exception of the sensitivity for cluster two, which was only 45.8%.

**Table 1:** Descriptive results per cluster (a) and results of cross-validation to test robustness of the clusters’ centers (b).

a	Cluster one	Cluster two	Cluster three
GCs (n)	181	59	190
Measurements (n)	91	17	45
Participants (n)	24	6	13

b	Reference membership		
	GCs in cluster one (n)	GCs in cluster two (n)	GCs in Cluster three (n)
Test membership: GCs in cluster one (n)	180	2	4
Test membership: GCs in cluster two (n)	1	27	0
Test membership: GCs in cluster three (n)	0	30	186
Sensitivity (%)	99.5%	45.8%	97.9%
Specificity (%)	97.6%	99.7%	87.5%
Misclassification rate (%) = 8.6%			

Number of GCs, measurements, and participants per cluster are given in (a). Confusion matrix between reference (i.e., cluster membership based on full dataset) and test memberships (i.e., predicted cluster membership after the 30-fold cross-validation) of the GCs with the sensitivity and specificity per cluster and overall misclassification rate are given in (b).

Abbreviations in alphabetic order: GCs = gait cycles; n = number.

### Aim 2: Characteristics of data-driven gait patterns

The medical and clinical background are summarized per cluster in Table S6 and are depicted per 3DGA-session of the boys with DMD in Figs. S4–S7. Details are provided online available at 10.48804/DQXROR.

#### Aim 2.a: Data-driven gait patterns compared to TD gait

**Figure 1:**
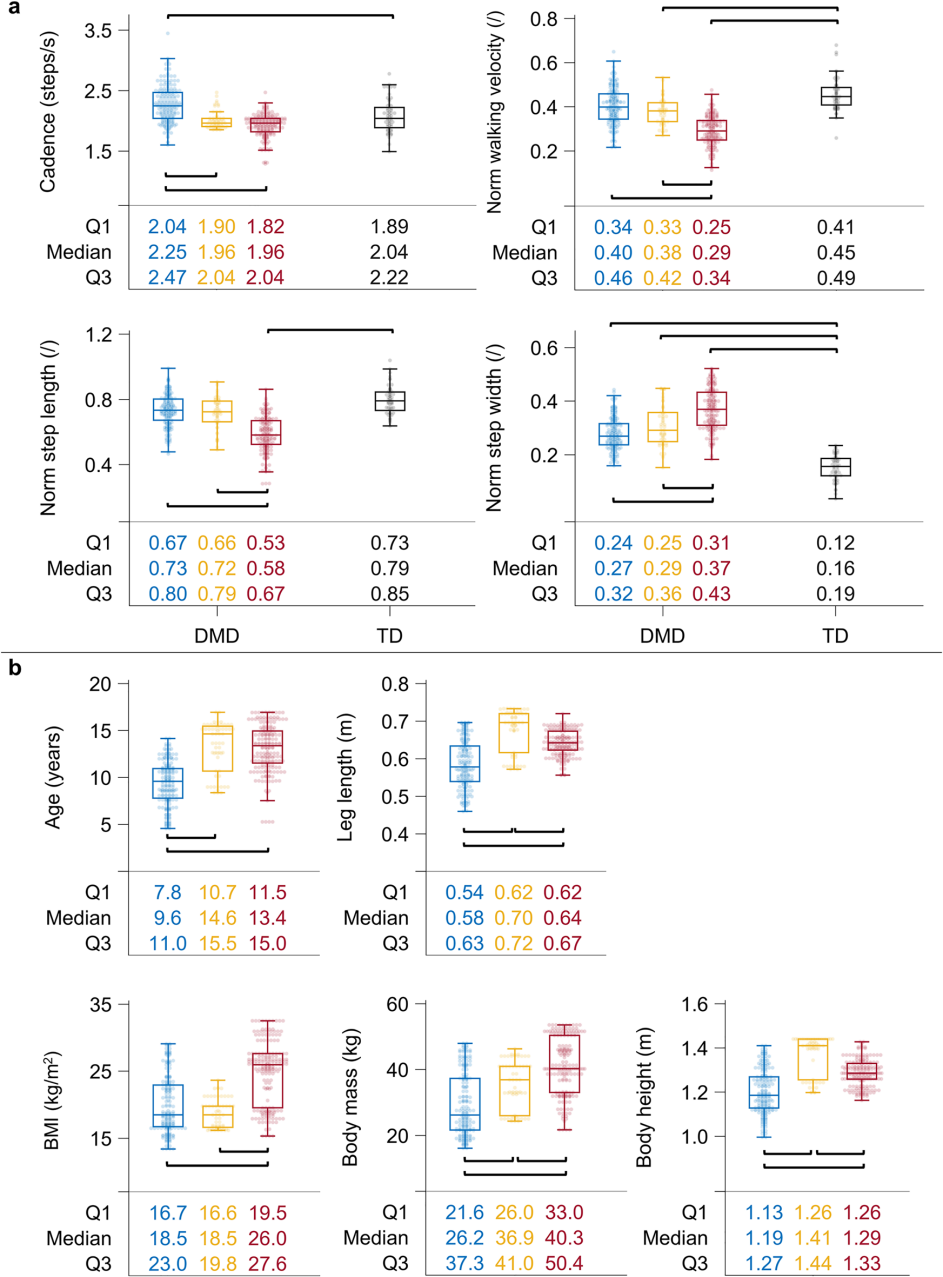
Spatiotemporal parameters of the clusters and TD children (**a**) and subject characteristics of the clusters (**b**). Box plots with individual data points and medians with Q1 and Q3 are visualized for cluster one (in blue), cluster two (in yellow), cluster three (in red) and TD children (in gray). The brackets indicate significant differences among groups that were indicated by the MWU tests (p < 0.0001). The results are visualized on the condition that (1) the KW tests (α = 0.006) revealed significant differences (for the comparison between the clusters) and (2) the results are clinically relevant, which was judged by the differences in spatiotemporal parameters between groups exceeding the previously reported MDC^45^ (i.e., a MDC of 0.135 for cadence after converting minutes to seconds, and a MDC of 0.068 for normalized step length, 0.034 for normalized step width and 0.058 for normalized walking velocity after normalizing to the average leg length and gravitational acceleration, according to the equations of Hof^31^). Abbreviations in alphabetic order: BMI = body mass index, DMD = Duchenne muscular dystrophy, kg = kilogram; KW = Kruskal–Wallis; m = meter; MWU = Mann–Whitney U; MDC = minimal detectable change; Norm = Normalized; Q1 = first quartile; Q3 = third quartile; TD = typically developing.

In comparison to TD, the cadence was larger in cluster one (p < 0.0001) (Fig. 1a). Normalized walking velocity was lower in clusters two and three, while normalized step length was only smaller in cluster three compared to TD (p < 0.0001). Normalized step width was larger in all three clusters compared to TD (p < 0.0001).

**Figure 2:**
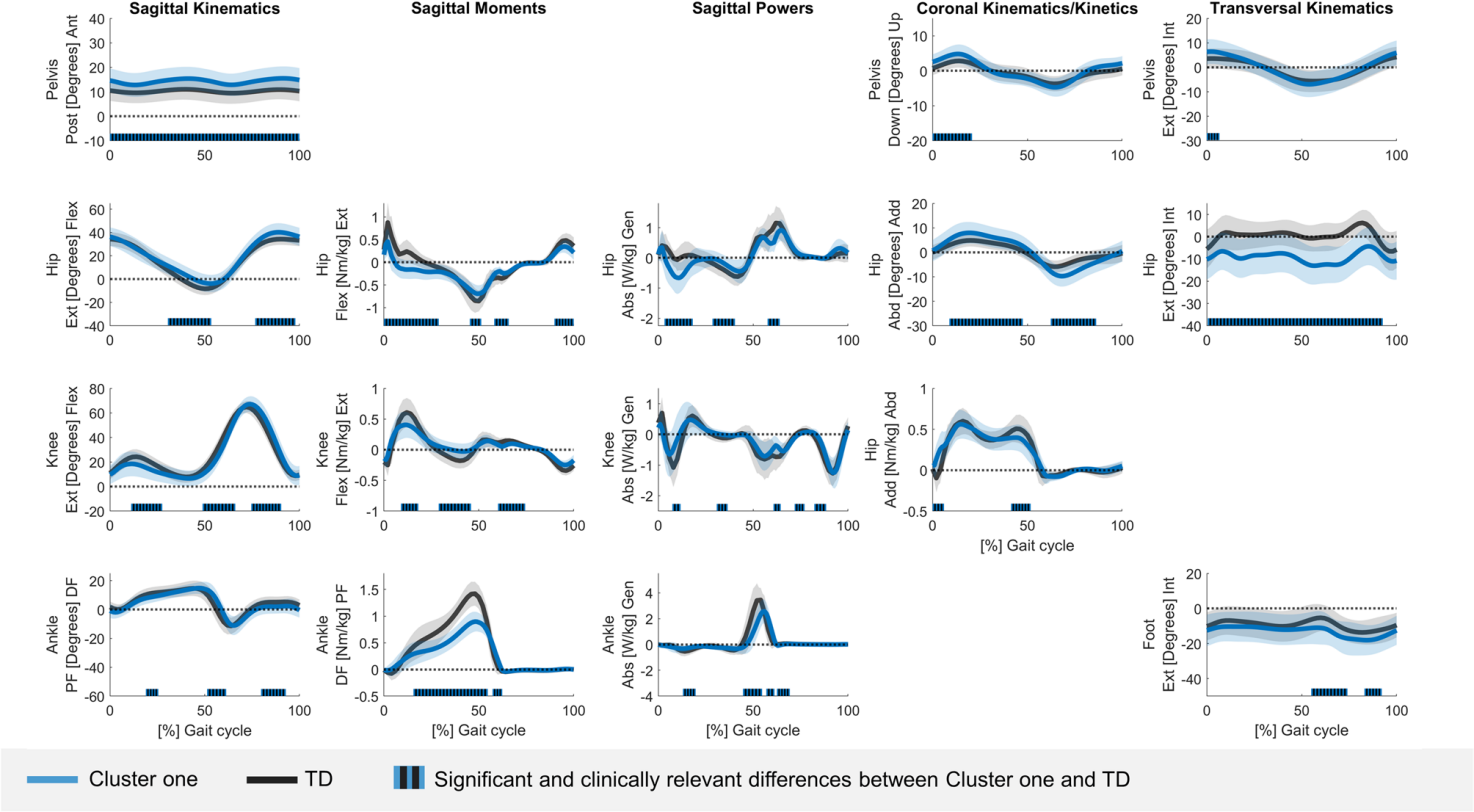
Gait pattern of cluster one compared to TD gait. The mean of the separate segment/joint waveforms with one standard deviation is visualized in blue for cluster one and in gray for TD children. The blocks (in blue/gray) on the x-axis represent the relevant and significant differences in waveforms (i.e., relevant suprathreshold clusters) between both gait patterns that were identified during the GC by the two-tailed t-test (α = 0.004–0.017). The results are visualized on the condition that (1) the Hotelling’s T^2^ test (α = 0.017) revealed significant differences on vector levels (sagittal, coronal and transversal kinematics, sagittal moments and powers) and (2) the results are clinically relevant, which was judged by the suprathreshold cluster’s duration being longer or equal to 3% of the GC and the differences in the segment/joint waveforms between both gait patterns exceeding the respective SEM, reported by Kainz et al.^38^, for 80% or more of the suprathreshold cluster’s duration. Abbreviations in alphabetic order: Abd = abduction; Abs = absorption; Add = adduction; Ant = anterior; Down = downwards; Ext in sagittal kinematics/kinetics = extension; Ext in Transversal kinematics = external; Flex = flexion; GC = gait cycle; Gen = generation; Int = internal; Nm/kg = Newton meter per kilogram; Post = posterior; SEM = standard error of measurement; TD = typically developing; Up = upwards; W/kg = Watt per kilogram.

**Figure 3:**
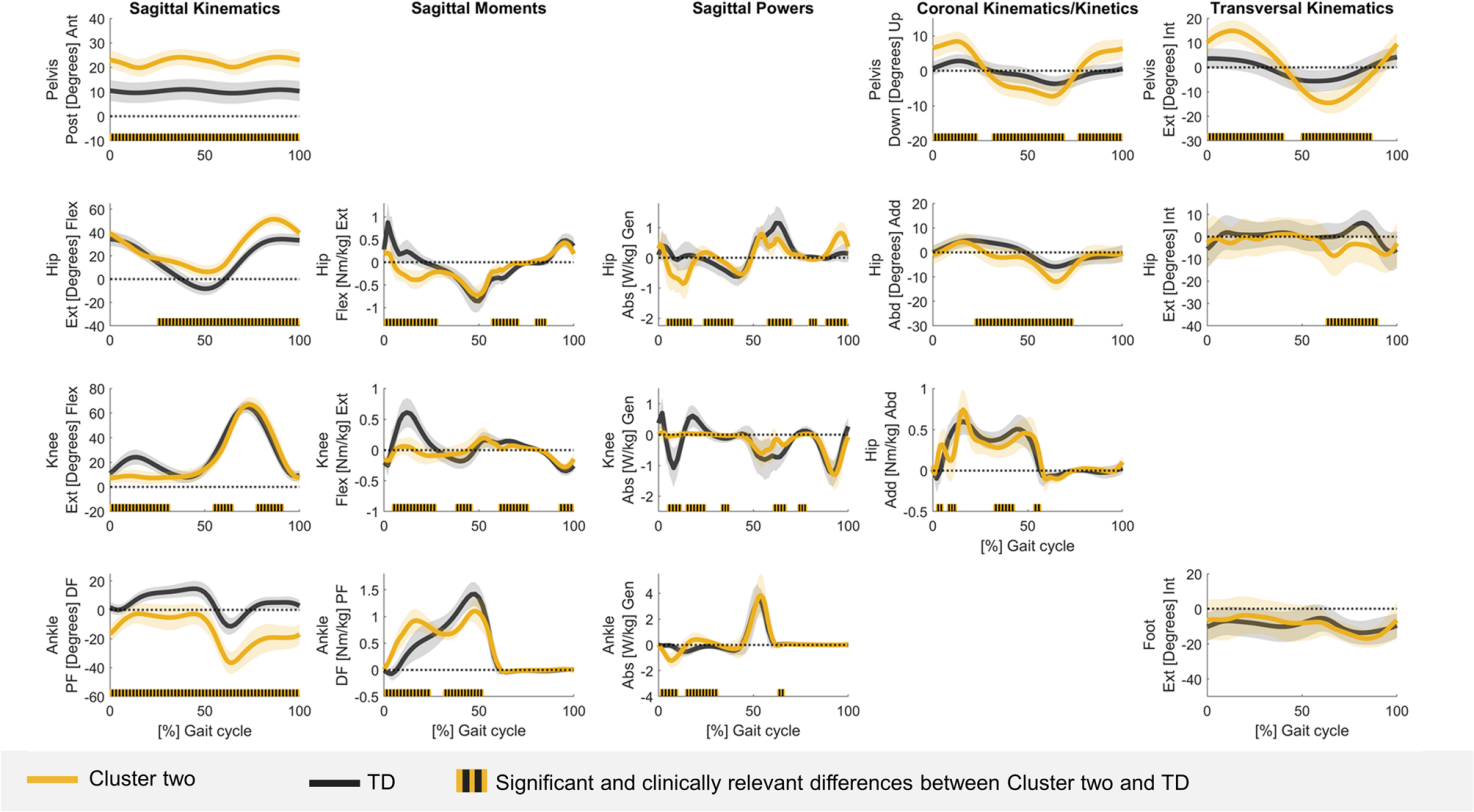
Gait pattern of cluster two compared to TD gait. The mean of the separate segment/joint waveforms with one standard deviation is visualized in yellow for cluster two and in gray for TD children. The blocks (in yellow/gray) on the x-axis represent the relevant and significant differences in waveforms (i.e., relevant suprathreshold clusters) between both gait patterns that were identified during the GC by the two-tailed t-test (α = 0.004–0.017). The results are visualized on the condition that (1) the Hotelling’s T^2^ test (α = 0.017) revealed significant differences on vector levels (sagittal, coronal and transversal kinematics, sagittal moments and powers) and (2) the results are clinically relevant, which was judged by the suprathreshold cluster's duration being longer or equal to 3% of the GC and the differences in the segment/joint waveforms between both gait patterns exceeding the respective SEM, reported by Kainz et al.^38^, for 80% or more of the suprathreshold cluster’s duration. Abbreviations in alphabetic order: Abd = abduction; Abs = absorption; Add = adduction; Ant = anterior; Down = downwards; Ext in sagittal kinematics/kinetics = extension; Ext in Transversal kinematics = external; Flex = flexion; GC = gait cycle; Gen = generation; Int = internal; Nm/kg = Newton meter per kilogram; Post = posterior; SEM = standard error of measurement; TD = typically developing; Up = upwards; W/kg = Watt per kilogram.

**Figure 4:**
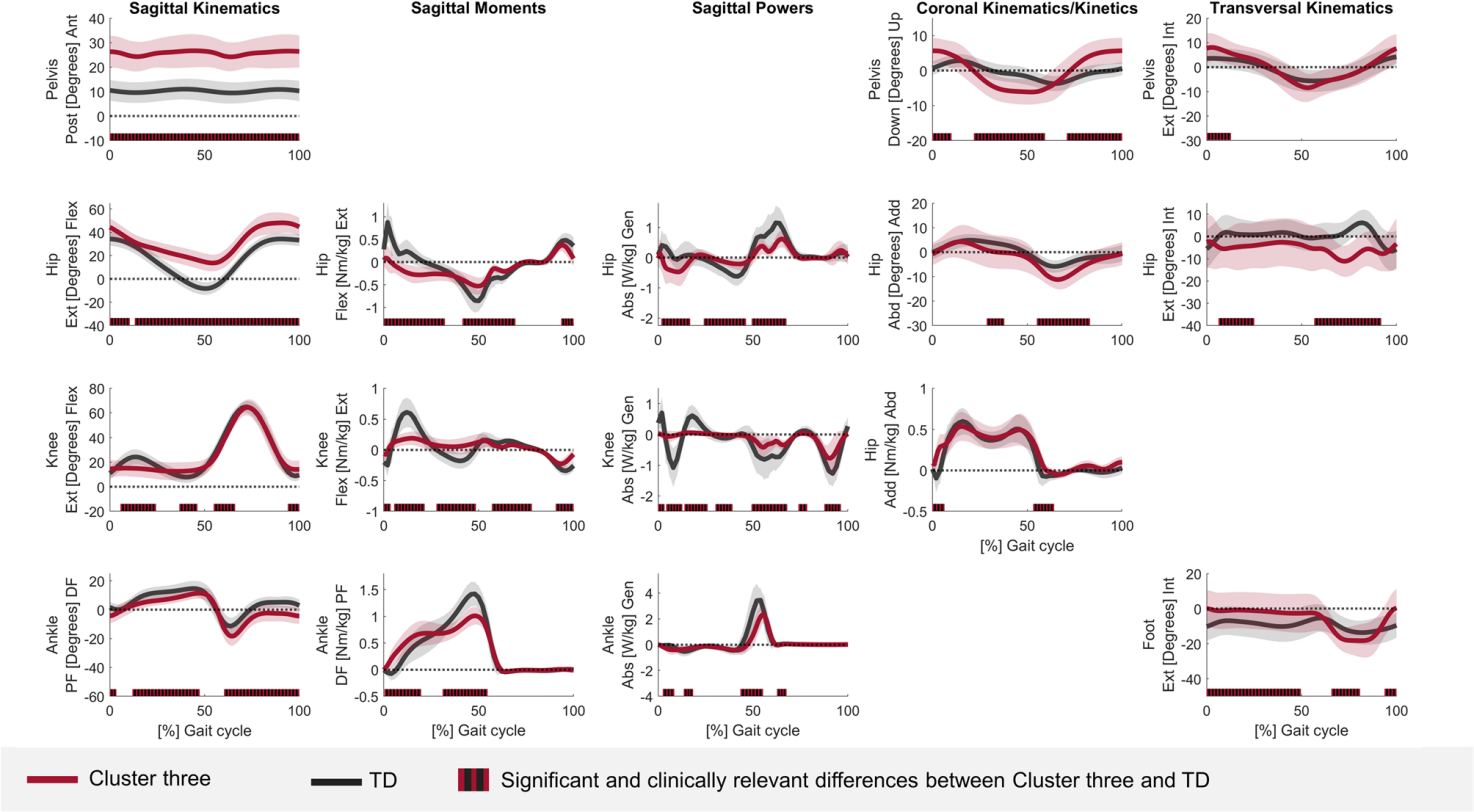
Gait pattern of cluster three compared to TD gait. The mean of the separate segment/joint waveforms with one standard deviation is visualized in red for cluster three and in gray for TD children. The blocks (in red/gray) on the x-axis represent the relevant and significant differences in waveforms (i.e., relevant suprathreshold clusters) between both gait patterns that were identified during the GC by the two-tailed t-test (α = 0.004–0.017). The results are visualized on the condition that (1) the Hotelling’s T^2^ test (α = 0.017) revealed significant differences on vector levels (sagittal, coronal and transversal kinematics, sagittal moments and powers) and (2) the results are clinically relevant, which was judged by the suprathreshold cluster's duration being longer or equal to 3% of the GC and the differences in the segment/joint waveforms between both gait patterns exceeding the respective SEM, reported by Kainz et al.^38^, for 80% or more of the suprathreshold cluster’s duration. Abbreviations in alphabetic order: Abd = abduction; Abs = absorption; Add = adduction; Ant = anterior; Down = downwards; Ext in sagittal kinematics/kinetics = extension; Ext in Transversal kinematics = external; Flex = flexion; GC = gait cycle; Gen = generation; Int = internal; Nm/kg = Newton meter per kilogram; Post = posterior; SEM = standard error of measurement; TD = typically developing; Up = upwards; W/kg = Watt per kilogram.

**Figure 5:**
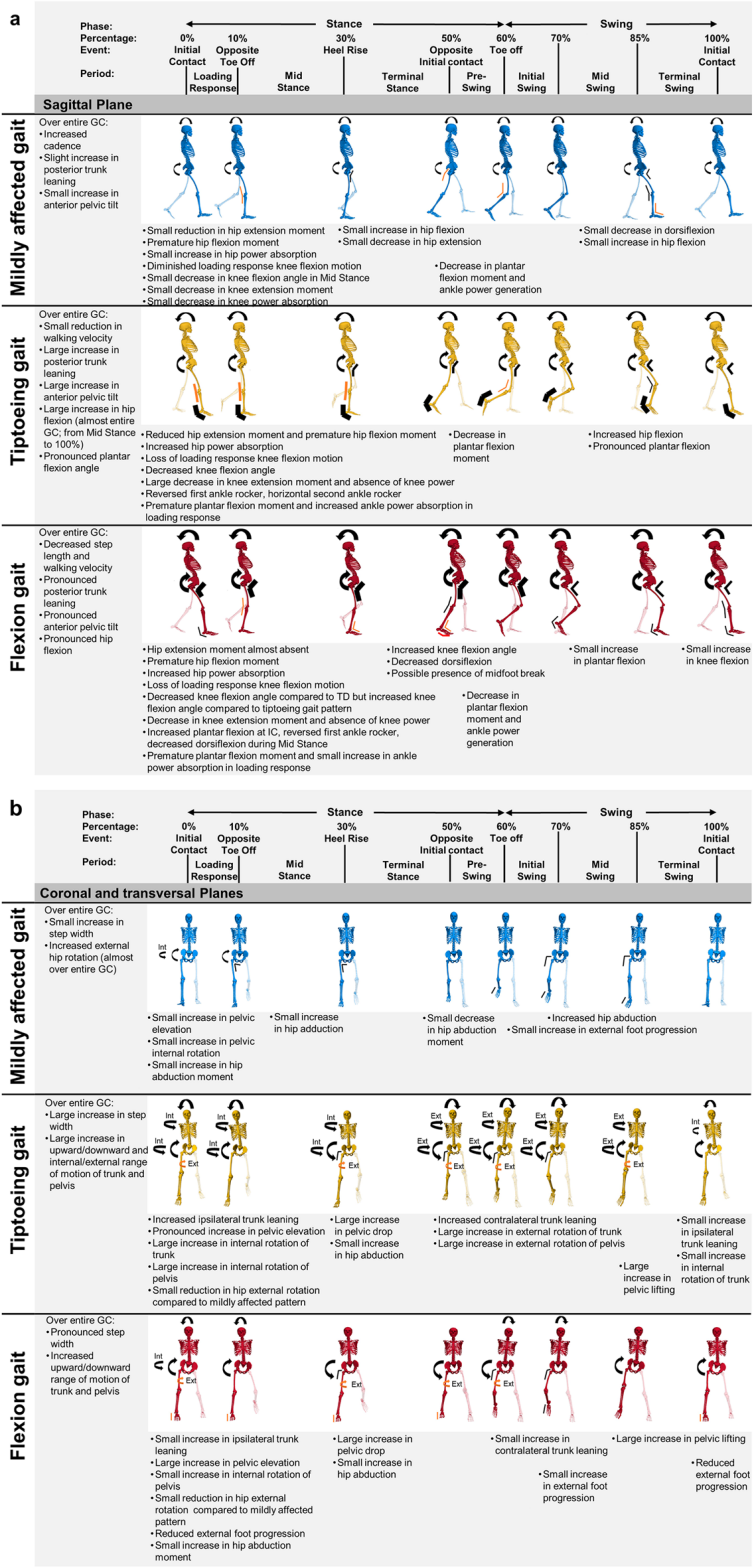
Overview of the gait features of the mildly affected gait pattern (in blue), the tiptoeing gait pattern (in yellow) and the flexion gait pattern (in red) in the sagittal plane (**a**) and the coronal and transversal planes (**b**). The symbols (arrows, angles, lines) illustrate the gait deviations. Their thickness represents the severity of the gait deviation, where thicker symbols indicate increased severity. The black color represents an increase in the respective angle, while the orange color represents a decrease in the respective angle. The red angle illustrates the presence of a midfoot break. Abbreviations in alphabetic order: GC = gait cycle; Ext = external; Int = Internal.

The kinematic and kinetic vectors of each cluster deviated from TD data for a large part of the GC (Tables S7–S9). The sum of all sections (in %) in the GC that differed from TD data ranged from 74.9 to 100% for the different vectors of cluster one, from 83.2 to 100% for the different vectors of cluster two, and from 88.6 to 100% for the different vectors of cluster three. At the segment/joint level, the gait pattern of cluster one only showed mild deviations from TD gait and was therefore labelled as the mildly affected gait pattern (Fig. 2/Table S7). Clusters two and three demonstrated similar deviations from TD gait as for the mildly affected gait pattern, but these deviations were more pronounced (Figs. 3, 4/Tables S8, S9). Additionally, the gait pattern of cluster two was characterized by a continuous plantar flexion angle during the entire GC, a premature plantar flexion moment and an increased ankle power absorption during loading response, and was therefore labelled as the tiptoeing gait pattern (Fig. 5). On the other hand, the gait pattern of cluster three was characterized by a distinct anterior pelvic tilt angle, an increased hip flexion angle and an increased knee flexion angle during terminal stance and was therefore labelled as the flexion pattern (Fig. 5).

#### Aim 2.b: Differences among data-driven gait patterns

The boys in cluster one were younger than the boys in clusters two and three (p < 0.0001), while age did not differ between clusters two and three (p = 0.4450) (Fig. 1b). In contrast, the BMI was similar in clusters one and two (p = 0.1339), while in cluster three the BMI was larger than in clusters one and two (p < 0.0001).

The cadence was larger in cluster one compared to clusters two and three (p < 0.0001). In cluster three, normalized walking velocity was lower, normalized step length was smaller and normalized step width was larger compared to clusters one and two (p < 0.0001) (Fig. 1a).

**Figure 6:**
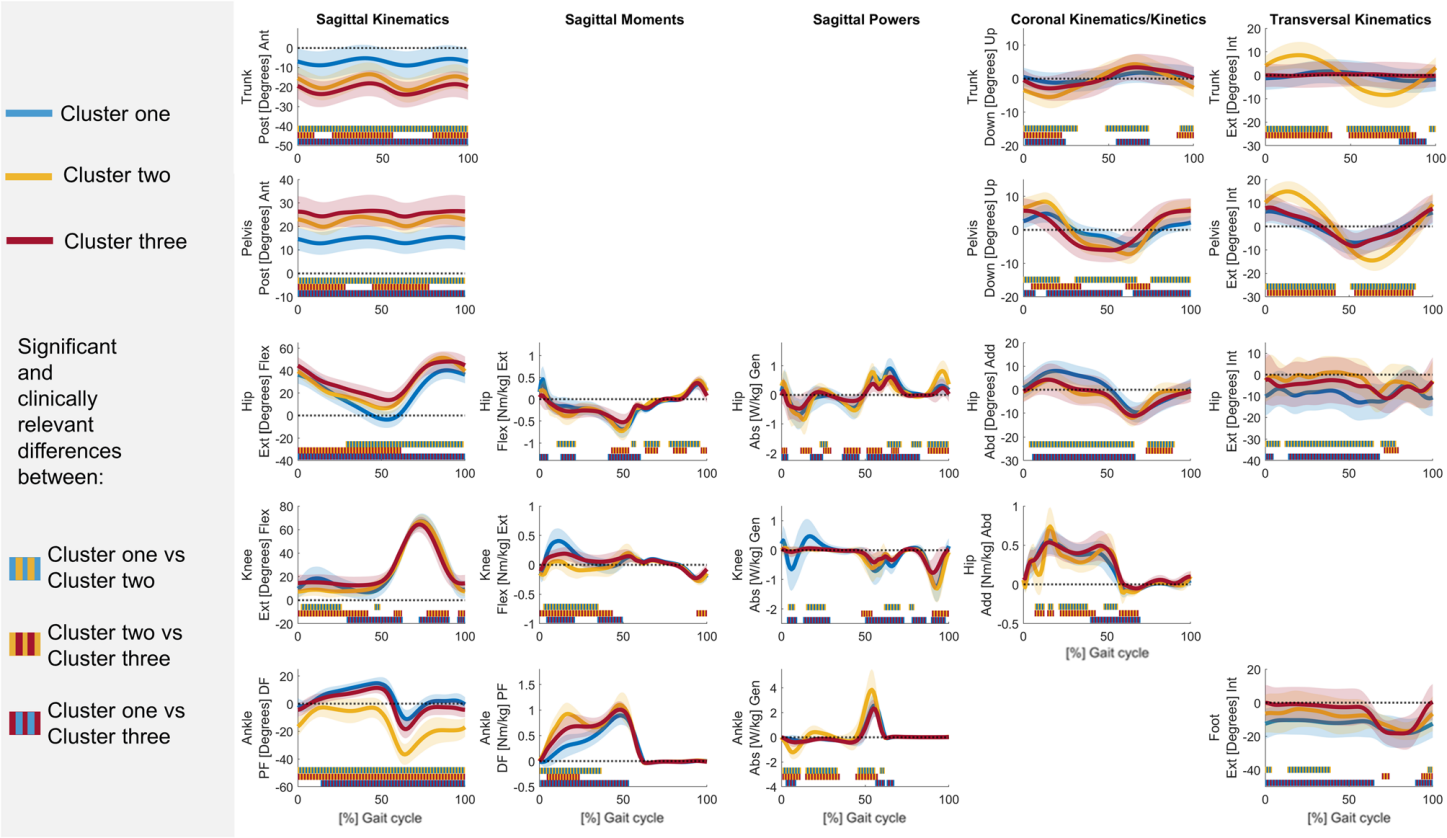
Gait patterns of clusters one, two and three. The mean of the separate segment/joint waveforms with one standard deviation is visualized in blue, yellow and red for cluster one, two and three, respectively. The blocks (in blue/yellow, yellow/red and blue/red gray for the comparison between clusters one and two, clusters two and three, and clusters one and three, respectively) on the x-axis represent the relevant and significant differences in waveforms (i.e., relevant suprathreshold clusters) among clusters that were identified during the GC by the two-tailed t-test (α = 0.003–0.017). The results are visualized on the condition that (1) the MANOVA (α = 0.05) and the Hotelling’s T^2^ test (α = 0.017) revealed significant differences on vector levels (sagittal, coronal and transversal kinematics, sagittal moments and powers) and (2) the results are clinically relevant, which was judged by the suprathreshold cluster's duration being longer or equal to 3% of the GC and the differences in the segment/joint waveforms between both gait patterns exceeding the respective SEM, reported by Kainz et al.^38^ and Wilken et al.^46^, for 80% or more of the suprathreshold cluster’s duration. Abbreviations in alphabetic order: Abd = abduction; Abs = absorption; Add = adduction; Ant = anterior; Down = downwards; Ext in sagittal kinematics/kinetics = extension; Ext in Transversal kinematics = external; Flex = flexion; GC = gait cycle; Gen = generation; Int = internal; Nm/kg = Newton meter per kilogram; Post = posterior; SEM = standard error of measurement; TD = typically developing; Up = upwards; W/kg = Watt per kilogram.

The kinematic and kinetic vectors were different over the entire GC among the three clusters (MANOVA results; Table S10). These differences in vectors were present for a large part of the GC (Tables S11–S13). The sum of all sections (in %) in the GC that differed between clusters one and two ranged from 79.7 to 100% for the different vectors, between clusters two and three ranged from 82.4 to 100% for the different vectors, and between clusters one and three ranged from 87.7 to 100% for the different vectors. At the segment/joint level, cluster two demonstrated the largest plantar flexion angle, premature plantar flexion moment, ankle power absorption and generation, upwards/downwards and internal/external trunk and pelvis range of motion, and the smallest knee flexion angle during loading response and midstance, and knee extension moment compared to clusters one and three (Figs. 5, 6/Tables S11–S13). In contrast, the posterior trunk leaning angle, anterior pelvic tilt angle, hip flexion angle, and internal foot progression angle were the largest in cluster three compared to clusters one and two.

### Aim 3: Comparison with Sutherland et al.

According to the Sutherland’s classification, 262 GCs were classified into the early stage, 144 GCs into the transitional stage, and only 24 GCs into the late stage. The overall agreement between Sutherland’s classification and the current classification was 50.2% (Table 2).

**Table 2:**
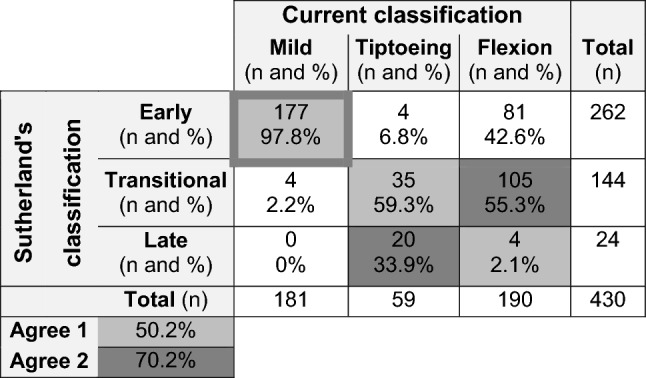
Contingency table and level of agreement between the current classification and the stages of Sutherland et al.

The number of GCs per gait pattern of our classification and stage of Sutherland et al. are given in counts (top of cells) as well as expressed in percentages relative to the total number of GCs classified into the mildly affected gait pattern (cluster one), tiptoeing gait pattern (cluster two) and flexion gait pattern (cluster three) (bottom of cells). The level of agreement was calculated in two ways. For the first approach, the overall agreement (Agree 1 in lighter gray) between both classifications was calculated, corresponding the early stage with the mildly affected gait pattern (cluster one), the transitional stage with the tiptoeing gait pattern (cluster two), and the late stage with the flexion gait pattern (cluster three). For the second approach, the overall agreement (Agree 2 in darker gray) between both classifications was calculated, corresponding the early stage with the mildly affected gait pattern (cluster one), the transitional stage with the flexion gait pattern (cluster three), and the late stage with the tiptoeing gait pattern (cluster two). Abbreviations in alphabetic order: Agree = agreement; GCs = gait cycles; n = number.

Since the gait patterns of the current classification did not fully align with increasing severity of gait deviations, we also calculated the overall agreement rate when Sutherland’s transitional stage would correspond to the flexion pattern (cluster three), and Sutherland’s late stage would correspond to the tiptoeing gait pattern (cluster two). For this second approach an overall agreement rate of 70.2% was found (Table 2).

## Discussion

We found three gait patterns in boys with DMD. These classes did not fully agree with previously introduced classifications^1,30^, which confirms the need for an updated classification system. The important difference with respect to previous classifications is that the gait patterns of the current classification did not fully align with increasing severity of gait deviations. We defined one mildly affected gait pattern and two more affected gait patterns, which differed based on the location of the most severe deviations, i.e. around the ankle for the tiptoeing gait pattern and around the proximal segments/joints for the flexion pattern. The discussion is further structured by specific research aim.

### Aim 1: to identify groups of similar patterns within the gait pathology

Hypothesis Aim 1.a was rejected, since only three data-driven gait patterns were distinguished in the gait pathology of children with DMD. Previous literature has also reported three gait patterns in children with DMD^1,30^.

Although three clusters were found in the current study, the clusters were not well separated and compact. This suggests that a continuum of gait patterns exists between cluster centers. This continuous change in gait deviations is not surprising, based on our previous longitudinal findings^26^. Due to the progressivity of the disorder, it is expected that patients would progress from the cluster’s center of a gait pattern to an inter-cluster boundary to the cluster’s center of a neighboring gait pattern. Thereby, patients would demonstrate stereotypical gait features of a gait pattern when they are close to that cluster’s center, then would present with features of a neighboring gait pattern as they move further away from the cluster’s center and closer to an inter-cluster boundary, and lastly would show stereotypical features of that neighboring gait pattern as they move closer to the cluster’s center of the neighboring gait pattern. Therefore, the period when the patients are close to the inter-cluster boundaries could be interpreted as “intermediate” stages. Future research in larger samples could calculate the distances to clusters’ centers and boundaries to quantify the progression through gait patterns and to identify intermediate stages.

Hypothesis Aim 1.b was confirmed, since the classification showed a high robustness (“Aim 1.b”). Therefore, the use of the clusters’ centers to classify GCs exhibited an overall good internal validity. Nonetheless, the sensitivity for cluster two was rather poor. It is worth noticing that cluster two was the smallest cluster, consisting of 59 GCs, but belonging to only six patients. Leaving one patient out (as part of the 30-fold cross-validation) strongly influenced the size of the cluster, and therefore, the cluster’s center could not be determined precisely, resulting in misclassification of the GCs. Nevertheless, the sensitivity and specificity of the other clusters were excellent.

### Aim 2: to describe and compare the characteristics of the data-driven gait patterns to TD children (Aim 2.a) and among the data-driven gait patterns (Aim 2.b)

Hypothesis Aim 2 was partially confirmed. Cluster one could be characterized as the mildly affected gait pattern, while clusters two (i.e., tiptoeing gait pattern) and three (i.e., flexion gait pattern) were characterized as the more advanced gait patterns.

In the mildly affected gait pattern, subtle deviations from TD gait were observed. A small increased anterior pelvic tilt in combination with less hip extension at the end of stance was seen. In contrast, the opposite (i.e., posterior pelvic tilt and hip hyperextension) has been reported in the early/mild group by previous gait classifications in DMD^1,30^. Yet, the anterior pelvic tilt of the control group (16°) in these studies was larger compared to the current study (10°), while a similar pelvic tilt is observed between the early stage of Sutherland et al.^1^ and the mildly affected gait pattern of the current study. Additionally, Doglio et al.^47^, who included a control group with a similar pelvic tilt compared to the current study, detected a trend towards a larger anterior pelvic tilt in young boys with DMD aged between 5 and 6.8 years old. For the remaining characteristics, similar minor deviations compared to previous literature were found^1,16,23,24,26,30,47–50^. As such, a slight increase in posterior trunk leaning was observed, as a compensation for hip extensor weakness^1^. Consequently, the hip extension moment reduced^26,48,50^, a premature hip flexion moment appeared^23,48^, and the hip power absorption increased. In agreement with Sienko Thomas et al.^30^, the knee angle over the entire GC approached the values of the knee angle in TD children. Yet, a minor decrease in knee flexion motion during loading response^24,30,48,49^ accompanied by a small decrease in knee extension moment^47–49^ and knee power absorption^48^, was detected. It is assumed that this subtle adaptation compensates for the commencing quadriceps weakness. While the ankle motion during stance was only slightly affected, the plantar flexion moment^26,47,48^ and ankle power generation during push-off^16,26,47,49^ were reduced, which may reflect weakness of the plantar flexors. During swing, a small increase in hip flexion probably helps clearing the foot, which shows a minimal drop foot^1,26^. Additionally, the hip abduction angle and the external foot progression were increased in swing^1,26^.

In the tiptoeing gait pattern, similar adaptations as in the mildly affected gait pattern were observed, but these adaptations were more advanced and the gait pathology around the ankle was the most prominent. The ankle angle was continuously in plantar flexion, with a reversed first rocker during loading response, a horizontal second rocker during midstance and terminal stance, and an increased plantar flexion angle during swing^1^. Consequently, a premature plantar flexion moment and increased ankle power absorption during loading response appeared^47^. At the knee, the knee flexion motion during loading was lost^48^. This resulted in the knee extension moment and knee power approaching zero, which reflects the diminished concentric and eccentric demand on the quadriceps muscle^48^. Another remarkable feature of this gait pattern was the increase in range of motion of the trunk and the pelvis in the coronal and transversal planes. In the coronal plane, a pattern of ipsilateral trunk leaning towards the supporting limb combined with contralateral pelvic lifting at the swinging limb, followed by an increased pelvic elevation at initial contact, was evident. This gait deviation has previously been reported^1,24,48,50^ and is considered to be a compensation mechanism for gluteus medius muscle weakness^1,48,50^. In the transversal plane, the increased motion in internal/external rotation of the pelvis and trunk may be a useful compensation strategy to steal step length^26,47^.

Also for the flexion gait pattern, the same adaptations as for the mildly affected gait pattern were observed, but these adaptations were even more pronounced and the marked hip and knee flexion in combination with distinct posterior trunk leaning and anterior pelvic tilt were the most prominent features. In contrast to the tiptoeing gait pattern, less deviations from TD gait were identified at the ankle. However, it is worth noticing that some of the boys in the flexion pattern presented with a midfoot break, which may have resulted in a dorsiflexion overestimation due to the limited number of markers on the foot. Similar as in the tiptoeing gait pattern, the boys in the flexion pattern showed the compensation strategy of lateral trunk bending and pelvis lifting^1,24,48,50^. Contrary, the compensation strategy of increased range of rotation motion in the trunk and pelvis was absent in the flexion pattern, which may explain the reduced step length^47^. Lastly, a distinct feature of the flexion pattern was the increased internal foot progression in stance. So far, this mechanism is not well understood^1^. It might potentially be related to increased stiffness and contractures of the hip abductors^26^.

### Aim 3: Comparison with Sutherland et al.

Hypothesis Aim 3 was confirmed due to the high overall disagreement rate between the current novel classification and Sutherland’s classification. While there was a high agreement between the mildly affected pattern and Sutherland’s early stage, the agreement between the two more advanced gait patterns in the current study and Sutherland’s transitional and late stages was rather low^1^. Additionally, only a low number of GCs was assigned to the late stage of Sutherland, further confirming that the progressive gait pathology of boys with DMD who received the current state-of-the-art medical care has not only been delayed, but has also changed.

The important difference between the current classification and the one of Sutherland et al.^1^ is that the current gait patterns did not fully correspond with increasing severity of gait deviations. Specifically, in addition to one mildly affected gait pattern, we identified two more affected gait patterns, which differed based on the location of the most severe deviations, i.e., around the ankle for the tiptoeing gait pattern and around the proximal segments/joints for the flexion gait pattern. Yet, in some boys in the flexion pattern, we observed an abnormally increased motion through the midfoot (i.e., midfoot break), resulting in a reduced effectiveness of the foot lever arm. Hence, the main difference between the tiptoeing and flexion pattern could be related to this foot lever arm deficit. This discrepancy was also reflected in the difference in age: the boys in the mildly affected gait pattern were young, whereas the boys in the two more affected gait patterns had similar ages (Fig. 1). In contrast, the BMI of the boys in the flexion pattern was higher than that of the boys in the tiptoeing gait pattern (Fig. 1). This suggests that the gait pathology in DMD is influenced not only by age (the natural decline due to progressive muscle weakness) but also by BMI.

The boys with DMD included in Sutherland’s classification had not received any medical interventions, and therefore, it is still an important natural history study. Due to the improved state-of-the-art medical care, this natural history has changed and the progressive gait pathology has not only been delayed, but has also changed. Yet, the discrepancies in medical and clinical background among the different boys with DMD in the current study (Table S6, Figs. S4–S7) resulted in a heterogeneous study sample. Therefore, there is an increased need for large-scale longitudinal multicenter studies with consistent medical interventions.

Since Sutherland et al.^1^, the nighttime wear of ankle foot orthoses and serial casting has been included in the clinical care to control for the development of gastrocnemius contractures^2,5,8^. The boys in the mildly affected and flexion gait patterns showed a good adherence to the nighttime ankle foot orthoses and the serial casting occurrence was only minimal (Table S6). This may explain the currently observed less severe progressive gait pathology at the ankle, compared to Sutherland et al.^1^ In contrast, the boys in the tiptoeing gait pattern showed a good adherence to the nighttime ankle foot orthoses only for 35.7% of the 3DGA-sessions and the serial casting occurrence was more frequent (28.6%). This suggests that the development of gastrocnemius contractures may not have been well controlled in this small group, which could have contributed to the development of a severe tiptoeing gait pattern.

To date, the corticosteroids-induced weight gain^51,52^ is an important issue in DMD. The boys, who walked with the flexion pattern and less deviations at the ankle, had a high BMI. Specifically, the BMI of these boys exceeded 25 kg/m^2^ for 52.4% of the sessions. In comparison, this percentage was 16.5% for the boys with the mildly affected gait pattern and 0% for the boys with the tiptoeing gait pattern (Table S6). Excess body mass therefore presumably plays an important role in affecting gait in DMD. A systematic review showed strong evidence for increased internal/external pelvis range of motion, increased hip internal rotation, and increased moments and powers at the hip, knee and ankle, and moderate evidence for increased step width in TD children with overweight or obesity compared to normal-weight peers^53^. In order to ensure stability and forward progression, it has been suggested that excess body mass requires muscles to produce higher moments^54^. Unlike for TD children, who can adjust to an increase in body mass by using muscles more effectively and increasing muscle strength^54^, the progressive muscle weakness in boys with DMD limits the ability to cope with increased load. Besides excess body mass, muscle contractures could impose an additional strain that necessitates even more muscle strength to ensure forward progression. Moreover, excess body mass may also result in flexible or rigid deformities. As such, more flexible feet with larger arch drops and lower arch rigidity when bearing weight have been previously reported in pediatric obesity^55^. This, in combination with gastrocnemius contractures restricting the ankle dorsiflexion^56^, are considered the likely causes of an abnormally increased motion through the midfoot (i.e., a midfoot break, examples are provided in Fig. S8), which could not be captured with the applied simplistic foot model. Yet, a midfoot break was indeed observed on the videos in some of the boys. Additionally, there appears to be a link with increasing BMI (Fig. S5). There was no discernible sequela from serial casting that could contribute to the midfoot break; however, nocturnal splinting may have potentially exerted an influence (Fig. S4). A midfoot break in DMD has never been reported before and is rather an unexpected finding. Usually, the tibilias posterior is contracted, and an equinovarus deformity is present^2^, which prevents eversion and increased dorsiflexion through the midfoot. It is possible that the overpull/contracture of the tibilias posterior develops in a later phase of the disease, when boys with DMD are spending less time bearing weight (more time sitting in a wheelchair)^2^ and therefore, the equinovarus deformity may be reported more frequently in non-ambulatory boys. Nutritional care aiming at the prevention of overweight and obesity may have the potential to improve gait pathology in DMD.

The added value of our results compared to previous studies^23,30^ lies in our comprehensive mapping and comparison of all and entire kinematic and kinetic waveforms across gait patterns, as well as with the gait pattern of TD children. Additionally, trunk kinematics, which had previously been lacking, were included in our analyses. Consequently, our findings provide more detailed insights than previous studies that focused solely on a single global functional outcome (such as 6MWT and NSAA), global gait parameters (such as the GDI)^30^, or specific gait parameters at certain points in the GC^23^. As a result, insights into the DMD gait pathology has been enhanced. Additionally, the link of the current classification with the scores of the NSAA and 6MWT highlights its clinical relevance (Figs. S6, S7). Boys with a flexion pattern tend to have lower NSAA scores and cover shorter distances in the 6MWT compared to those with the other two gait patterns. Moreover, some boys exhibiting the flexion pattern lost ambulation shortly after their last measurement session (Fig. S4). These findings emphasize the clinical importance of steering boys with DMD away from the flexion pattern and focusing on addressing the midfoot break. However, it is premature to state that the current classification is more clinically useful than findings from previous studies^23,30^ due to the limited number of participants in the more severely affected gait patterns. Additionally, there was overlap between the two more affected gait patterns. It is therefore not yet clear whether the tiptoeing gait pattern is a valid separate gait pattern or whether the tiptoeing and flexion gait patterns should be considered as one more affected gait pattern. This highlights the complexity of the continuous changes in gait patterns, especially during the late phases. Therefore, future studies with a longer follow-up until loss of ambulation and a larger number of more severely affected boys are needed to verify the sensitivity of our findings, prior to the transfer to the daily clinic. Nevertheless, a classification based on detailed gait data (across entire waveforms) may ultimately offer greater utility and potential than classifications relying solely on single outcome measures.

### Preliminary longitudinal explorations

The longitudinal evolution from gait pattern to gait pattern within the participants was explored in Fig. S4. Overlap between gait patterns for a particular 3DGA-session was displayed by coloring the markers in the figure. The classification of the session was interpreted according to the majority of the GCs’ assignments. However, these sessions with overlap could also indicate an “intermediate” stage between gait patterns. Twenty-one boys consistently demonstrated the same gait pattern during their follow-up and did thus not show any transition to another gait pattern. Specifically, 16 patients stayed in the mildly affected gait pattern, four patients in the flexion pattern, and one patient in the tiptoeing gait pattern. Transitions from the mildly affected to the flexion gait pattern were detected in five patients. Three of these five patients showed a period where they were assigned to both the mildly affected and flexion gait patterns, which could indicate an “intermediate” stage. Good adherence to nighttime ankle foot orthoses, almost no serial casting (except for one patient), and consistent participation in clinical trials with disease-modifying medication (in four patients) were reported for this group. Additionally, this transition was often observed in case of increasing BMI (Fig. S5; especially in four patients). Two patients transitioned from the mildly affected to the tiptoeing gait pattern. Interestingly, these participants did not adhere to the nighttime ankle foot orthoses. One of them received serial casting twice and participated in a clinical trial with disease-modifying medication. One patient (with good adherence to nighttime ankle foot orthosis and participation in clinical trial with disease-modifying medication) first showed a transition from the mildly affected to the flexion gait pattern and then demonstrated both the tiptoeing and the flexion gait patterns. Lastly, a deviant trajectory was detected for one patient (with nonadherence to nighttime ankle foot orthosis, no participation in clinical trial with disease-modifying medication), who first transitioned from the flexion to the tiptoeing gait pattern and then demonstrated both the tiptoeing and flexion gait patterns. However, overlap was detected for all of his sessions, which could indicate an “intermediate” stage between these two gait patterns. The causes of the transitions cannot be deduced from the medical and clinical background of the participants. The current data suggest that a transition to the tiptoeing gait pattern occurs in case of nonadherence to the nighttime ankle foot orthosis, while a transition to the flexion gait pattern occurs in case of increasing BMI. However, future research should further investigate how medical interventions and clinical background, as well as underlying impairments such as progressive muscle weakness and contractures, contribute to the progressive gait pathology in boys with DMD.

## Limitations

The inconsistencies in medical background among boys with DMD contributed to a large heterogeneity of our study sample (Table S6, Figs. S4–S7), hindering the generalization of the results and the ability to accurately determine the impact of contemporary interventions on the current DMD gait pathology. Longitudinal studies with consistent medical interventions would be of much value in the future. Our simplistic foot model could have resulted in a dorsiflexion overestimation. Therefore, a detailed description of the complex gait deviations around the foot and ankle is lacking in the current gait classification. Future research with more complex foot models in combination with radiographs and foot pressure studies are needed to further capture and investigate our unexpected finding of a midfoot break. Although a large longitudinal database was used, the sample size at the level of the individual participant for the two more affected gait patterns, especially for the tiptoeing gait pattern, was still limited and the age range among participants was wide. The current classification can therefore not yet be considered valid and sensitive for the entire DMD population. More boys need to be followed until ambulation is lost to support our findings. An external validation to test whether new patients from other pediatric centers for neuromuscular diseases in European countries could be classified according to the current classification system would be extremely valuable. Groups of similar gait patterns were identified based on a cross-sectional approach.

## Conclusion

This investigation found three gait patterns in DMD: a mildly affected, a tiptoeing and a flexion pattern. Their agreement with Sutherland’s classification^1^ was rather low, especially regarding the two more affected gait patterns, which suggests that the progressive gait pathology of boys with DMD who received the current state-of-the-art medical care has not only been delayed, but has also changed. The results of the current study improved insights into the gait pathology in children with DMD. However, the classes were not well separated and compact. Overlap between classes was especially seen between the two more affected classes, highlighting the complexity of the continuous changes in gait patterns, especially during the late phases. Therefore, caution is required when classifying individual children into classes.

### Supplementary Information

Supplementary Information.

## Data Availability

The datasets supporting the conclusions of this article are openly available in the Research Data Repository (RDR) of KU Leuven at 10.48804/DQXROR. The datasets are also available from the corresponding author on reasonable request.
