# Supplementary material for: Gait classification for growing children with Duchenne muscular dystrophy
Source: Sci Rep. 2024 May 11;14:10828. doi: 10.1038/s41598-024-61231-y (PMC11088636; doi:10.1038/s41598-024-61231-y)
Supplement: Supplementary file 1 — Supplementary Information. [file 41598_2024_61231_MOESM1_ESM.pdf]

Table S1: Overview of the extracted waveforms, spatiotemporal and discrete gait parameters.

**19 continuous waveforms**	**6 parameters**
Kinematics (°)	4 spatiotemporal parameters
Trunk *	Cadence (steps/s and steps/min)
Sagittal *	Normalized walking velocity (/)
Coronal *	Normalized step length (/)
Transversal *	Normalized step width (/)
Pelvis	
Sagittal	2 discrete kinematic parameters (°)
Coronal	Maximal anterior pelvic tilt in stance
Transversal	Maximal dorsiflexion angle in swing
Hip	
Sagittal	
Coronal	
Transversal	
Knee	
Sagittal	
Ankle	
Sagittal	
Foot	
Transversal	
Kinetic Moments (Nm/kg)	
Hip	
Sagittal	
Coronal	
Knee	
Sagittal	
Ankle	
Sagittal	
Kinetic Powers (W/kg)	
Hip	
Sagittal	
Knee	
Sagittal	
Ankle	
Sagittal	

*****Kinematic data of the trunk was only available for the boys with DMD (the Plug-In Gait Full-Body marker model was used in DMD and the Plug-In Gait Lower-Body was used in TD).
Abbreviations in alphabetic order: DMD=Duchenne muscular dystrophy; kg=kilogram; m=meter; min=minutes; Nm=Newton meter; s=seconds; TD=typically developing; W=Watt

Table S2: Overview of the extracted discrete parameters and criteria to classify gait cycles according to Sutherland's classification[1]

**Sutherland's classification**				
**Parameters**	**Formula**	**Early Stage**	**Transitional Stage**	**Late Stage**
Cadence (Cad, in steps/min)<br>Maximal anterior pelvic tilt angle in stance (PT, in degrees)<br>Maximal ankle dorsiflexion angle in swing (DF, in degrees)	L(x)= -0.252+0.032(Cad) -0.132(PT) +0.147(DF)	L(x) > 0	-3<L(x)<0	L(x) < -3

Sutherland's classification: for each GC, the values of the 3 parameters were filled in the formula. If the outcome of the formula was positive, negative (between 0 and -3) or smaller than -3, than the GC was classified into the early, transitional and late stage, respectively.

Abbreviations in alphabetic order: Cad=cadence; DF=maximum ankle dorsiflexion in swing; GC=gait cycle; L(x)=Fisher linear discriminant formula; min=minutes; PT=maximum pelvic tilt in stance; s=seconds

Table S3: Overview of the inputs for the PCAs and retained PCs per PCA.

**Inputs for PCAs**	**Retained PCs**
**4 spatiotemporal parameters (430 GCs x 4 parameters)**	2
Cadence (steps/s)	
Normalized walking velocity (/)	
Normalized step length (/)	
Normalized step width (/)	
17 continuous waveforms (430 GCs x 101 datapoints)	
Kinematics (°)	
Trunk	
**Sagittal**	1
**Coronal**	2
**Transversal**	2
Pelvis	
**Sagittal**	1
**Coronal**	3
**Transversal**	3
Hip	
**Sagittal**	2
**Coronal**	2
Knee	
**Sagittal**	3
Ankle	
**Sagittal**	1
Foot	
**Transversal**	2
Kinetic Moments (Nm/kg)	
Hip	
**Sagittal**	7
Knee	
**Sagittal**	5
Ankle	
**Sagittal**	6
Kinetic Powers (W/kg)	
Hip	
**Sagittal**	10
Knee	
**Sagittal**	8
Ankle	
**Sagittal**	10
	Total=70

Multiple PCAs were performed. The input per PCA is indicated in bold. One PCA was performed on the spatiotemporal parameters (430 GCs x 4 parameters). Seventeen out of the 19 extracted continuous waveforms (Table S1) were included in the PCAs. The waveform of the hip internal rotation angle and the waveform of the hip abduction moment were excluded from these analyses, because hip internal rotation angle is prone to error and the maximal hip abduction moment presented a complex longitudinal evolution. For each of the remaining 17 continuous waveforms, a separate PCA was performed (430 GCs x 101 data points, for 17 waveforms). The PCs that expressed a cumulative variability of 80% were retained. The number of retained PCs per PCA are documented in the right column.

Abbreviations in alphabetic order: GC=gait cycle; PCA=principal component analysis, PC=principal component

Table S4: Significant thresholds for the comparisons of kinematic and kinetic waveforms between the three clusters and typically developing children, according to the Bonferroni correction[2]

	Joints/segments	Significant threshold
Sagittal plane kinematics	1. Pelvis	0.05/(4 segments or joints * 3 pairwise comparisons) = 0.004
	2. Hip	0.05/(4 segments or joints * 3 pairwise comparisons) = 0.004
	3. Knee	0.05/(4 segments or joints * 3 pairwise comparisons) = 0.004
	4. Ankle	0.05/(4 segments or joints * 3 pairwise comparisons) = 0.004
Coronal plane kinematics	1. Pelvis	0.05/(2 segments or joints * 3 pairwise comparisons) = 0.008
	2. Hip	0.05/(2 segments or joints * 3 pairwise comparisons) = 0.008
Transversal plane kinematics	1. Pelvis	0.05/(3 segments or joints * 3 pairwise comparisons) = 0.006
	2. Hip	0.05/(3 segments or joints * 3 pairwise comparisons) = 0.006
	3. Foot	0.05/(3 segments or joints * 3 pairwise comparisons) = 0.006
Sagittal plane moments	1. Hip	0.05/(3 segments or joints * 3 pairwise comparisons) = 0.006
	2. Knee	0.05/(3 segments or joints * 3 pairwise comparisons) = 0.006
	3. Ankle	0.05/(3 segments or joints * 3 pairwise comparisons) = 0.006
Coronal plane moment	1. Hip	0.05/(1 segment or joint * 3 pairwise comparisons) = 0.017
Powers	1. Hip	0.05/(3 segments or joints * 3 pairwise comparisons) = 0.006
	2. Knee	0.05/(3 segments or joints * 3 pairwise comparisons) = 0.006
	3. Ankle	0.05/(3 segments or joints * 3 pairwise comparisons) = 0.006

The alpha level of 0.05 was corrected to consider the number of segments or joints per motion plane of the kinematics, moments and powers in three pairwise comparisons (i.e., cluster one vs typically developing children, cluster two vs typically developing children, and cluster three vs typically developing children), according to the Bonferroni correction[2].

Table S5: Significant thresholds for the comparisons of kinematic and kinetic waveforms between the three clusters, according to the Bonferroni correction[2]

	Joints/segments	Significant threshold
Sagittal plane kinematics	1. Trunk	0.05/(5 segments or joints * 3 pairwise comparisons) = 0.003
	2. Pelvis	0.05/(5 segments or joints * 3 pairwise comparisons) = 0.003
	3. Hip	0.05/(5 segments or joints * 3 pairwise comparisons) = 0.003
	4. Knee	0.05/(5 segments or joints * 3 pairwise comparisons) = 0.003
	5. Ankle	0.05/(5 segments or joints * 3 pairwise comparisons) = 0.003
Coronal plane kinematics	1. Trunk	0.05/(3 segments or joints * 3 pairwise comparisons) = 0.006
	2. Pelvis	0.05/(3 segments or joints * 3 pairwise comparisons) = 0.006
	3. Hip	0.05/(3 segments or joints * 3 pairwise comparisons) = 0.006
Transversal plane kinematics	1. Trunk	0.05/(4 segments or joints * 3 pairwise comparisons) = 0.004
	2. Pelvis	0.05/(4 segments or joints * 3 pairwise comparisons) = 0.004
	3. Hip	0.05/(4 segments or joints * 3 pairwise comparisons) = 0.004
	4. Foot	0.05/(4 segments or joints * 3 pairwise comparisons) = 0.004
Sagittal plane moments	1. Hip	0.05/(3 segments or joints * 3 pairwise comparisons) = 0.006
	2. Knee	0.05/(3 segments or joints * 3 pairwise comparisons) = 0.006
	3. Ankle	0.05/(3 segments or joints * 3 pairwise comparisons) = 0.006
Coronal plane moment	1. Hip	0.05/(1 segment or joint * 3 pairwise comparisons) = 0.017
Powers	1. Hip	0.05/(3 segments or joints * 3 pairwise comparisons) = 0.006
	2. Knee	0.05/(3 segments or joints * 3 pairwise comparisons) = 0.006
	3. Ankle	0.05/(3 segments or joints * 3 pairwise comparisons) = 0.006

The alpha level of 0.05 was corrected to consider the number of segments or joints per motion plane of the kinematics, moments and powers in three pairwise comparisons (i.e., cluster one vs cluster two, cluster two vs cluster three, and cluster three vs cluster one), according to the Bonferroni correction[2].

Table S6: Medical and clinical background per cluster

	Cluster 1	Cluster 2	Cluster 3
BMI < 18.5 (% of sessions)	46.2	64.3	14.3
BMI >= 18.5 & < 25 (% of sessions)	37.4	35.7	33.3
BMI >= 25 & < 30 (% of sessions)	16.5	0.0	42.9
BMI >= 30 (% of sessions)	0.0	0.0	9.5
Deflazacort (% of sessions)	96.7	100.0	97.6
Vamorolone (% of sessions)	1.1	0.0	2.4
No steroids (% of sessions)	2.2	0.0	0.0
Participation Clinical Trial with disease-modifying medication (% of sessions)	28.6	64.3	66.7
Adherence nighttime AFO (% of sessions)	78.0	35.7	90.5
Serial Casting (% of sessions)	6.6	28.6	2.4
Loss of ambulation (% of participants)	4.2	80.0	63.6
NSAA (median)	28.0	21.0	14.0
6MWT (median)	374.5	414.5	246.0

The frequencies of the medical and clinical background at the session level (expect for loss of ambulation, which was reported at the participation level) are provided per cluster. For the NSAA and 6MWT, the median value is reported per cluster.
Abbreviations in alphabetic order: 6MWT=6-min walk test; BMI=body mass index; NSAA=North Star Ambulatory Assessment

Table S7: Kinematic and kinetic differences between the mildly affected gait pattern (i.e., cluster one) and typically developing children.

		n	T2*/t*	Suprathreshold cluster 1 % extent (range)	Suprathreshold cluster 2 % extent (range)	Suprathreshold cluster 3 % extent (range)	Suprathreshold cluster 4 % extent (range)	Suprathreshold cluster 5 % extent (range)	SEM [3]
Sagittal kinematics	Vector[a]	1	19.48	100 (0-100)					
	Pelvis[d]	1	3.17	100 (0-100)					2.70
	Hip[d]	2	3.51	22.9 (30.6-53.5)	21.2 (76.7-97.9)				2.80
	Knee[d]	3	3.72	16.4 (11.2-27.6)	17.3 (48.9-66.2)	15.8 (74.6-90.4)			2.30
	Ankle[d]	3	3.89	6.6 (18.9-25.5)	10.1 (51.3-61.3)	13.1 (79.7-92.8)			2.00
Coronal kinematics	Vector[a]	2	13.78	55.7 (0-55.7)	37.7 (62.3-100)				
	Pelvis[b]	1	3.39	20.8 (0-20.8)					1.70
	Hip[b]	2	3.55	38.7 (8.9-47.6)	23.9 (62.3-86.2)				2.50
Transversal kinematics	Vector[a]	1	15.87	100 (0-100)					
	Pelvis[c]	1	3.49	6.6 (0-6.6)[e]					1.80
	Hip[c]	1	3.55	92.2 (0.5-92.7)					4.70
	Foot[c]	2	3.50	19.1 (55.0-74.1)	9.1 (83.1-92.2)				/
Sagittal Moments	Vector[a]	2	20.03	54.9 (0-54.9)	42.9 (57.1-100)				
	Hip[c]	4	3.84	28.9 (0-28.9)	6 (45.2-51.3)	7.7 (58.1-65.8)	10.2 (89.8-100)		0.05
	Knee[c]	3	3.94	9.1 (9-18.1)	17.1 (28.8-45.9)	14.3 (60.1-74.4)			0.05
	Ankle[c]	2	3.82	39.2 (15.5-54.7)	5.1 (57.2-62.3)				0.05
Coronal Moment	Hip[a]	2	3.54	6.1 (0-6.1)	10.3 (41.4-51.7)				0.05
Powers	Vector[a]	5	20.91	19.3 (1.3-20.6)	13.5 (27.0-40.5)	10.1 (44.7-54.7)	21.2 (56.7-77.9)	10.8 (82.0-92.9)	
	Hip[c]	3	3.88	14.9 (3.3-18.2)	11.9 (28.6-40.4)	6.4 (57.6-64.0)			/
	Knee[c]	5	4.17	4.3 (7.4-11.8)	5.7 (30.8-36.6)	3.8 (60.7-64.5)	5.2 (71.9-77.1)	6.2 (82.3-88.5)	/
	Ankle[c]	4	3.96	6.7 (13.0-19.7)	10.1 (44.7-54.7)	4.3 (57.0-61.2)	6.8 (62.5-69.3)		/

[a] $\alpha=0.017$; [b] $\alpha=0.008$; [c] $\alpha=0.006$; [d] $\alpha=0.004$

all $p \leq 0.001$ apart from [e] $p = 0.0019$

All relevant and significant suprathreshold clusters that were identified during the GC by the Hotteling's T^2 test for the vectors (i.e., components consisting of the combination of the individual motions per plane and type of waveform: sagittal kinematics, coronal kinematics, transversal kinematics, sagittal moments, powers) and the two-tailed t-tests for the segment or joint waveforms, are reported. The later are reported on the condition that (1) the Hotelling's T^2 test revealed significant differences on vector levels and (2) the results are clinically relevant, which was judged by the suprathreshold cluster's duration being longer or equal to 3% of the GC and the differences in the segment or joint waveforms between both gait patterns exceeding the respective SEM for the intra-rater intersession reliability in TD, reported by Kainz et al.[3], for 80% or more of the suprathreshold cluster's duration.

GC=gait cycle; n=number of identified suprathreshold clusters; SEM=standard error of measurement; T2*/t*=critical threshold needed to reject the null hypothesis; % extent (range)=extent of the identified suprathreshold cluster (start and end points of the suprathreshold cluster)

Table S8: Kinematic and kinetic differences between the tiptoeing gait pattern (i.e., cluster two) and typically developing children.

		n	T2*/t*	Suprathreshold cluster 1 % extent (range)	Suprathreshold cluster 2 % extent (range)	Suprathreshold cluster 3 % extent (range)	Suprathreshold cluster 4 % extent (range)	Suprathreshold cluster 5 % extent (range)	SEM[3]
Sagittal kinematics	Vector[a]	1	18.32	100 (0-100)					
	Pelvis[d]	1	3.29	100 (0-100)					2.70
	Hip[d]	1	3.50	75.2 (24.8-100)					2.80
	Knee[d]	3	3.70	31.9 (0-31.9)	11.3 (54.1-65.3)	15.1 (76.7-91.8)			2.30
	Ankle[d]	1	3.45	100 (0-100)					2.00
Coronal kinematics	Vector[a]	1	13.63	100 (0-100)					
	Pelvis[b]	3	3.52	23.8 (0-23.8)	38.7 (31.0-69.7)	23.8 (76.2-100)			1.70
	Hip[b]	1	3.41	52.5 (21.8-74.3)					2.50
Transversal kinematics	Vector[a]	3	15.81	41.1 (0-41.1)	41.9 (49.2-91.1)	2.4 (97.6-100)[e]			
	Pelvis[c]	2	3.45	40.8 (0-40.8)	38.1 (49.2-87.3)				1.80
	Hip[c]	1	3.60	28.2 (62.3-90.5)					4.70
	Foot[c]	0	3.48						/
Sagittal Moments	Vector[a]	2	19.07	52.8 (0-52.8)	44.8 (55.2-100)				
	Hip[c]	3	4.00	28.6 (0-28.6)	15.1 (56.3-71.4)	6.7 (79.2-85.8)			0.05
	Knee[c]	4	3.87	23.7 (4-27.8)	9.4 (37.4-46.8)	16.3 (60.2-76.5)	8.1 (91.9-100)		0.05
	Ankle[c]	2	3.73	24.7 (0.1-24.7)	21.4 (31.1-52.5)				0.05
Coronal Moment	Hip[a]	4	3.57	4.0 (1.6-5.7)	5.2 (7.5-12.7)	11.5 (31.9-43.4)	4.4 (52.8-57.2)		0.05
Powers	Vector[a]	3	19.25	40 (0-40)	30.3 (54.5-84.8)	12.8 (87.2-100)			
	Hip[c]	5	3.94	14.3 (3.9-18.1)	16.6 (23.3-39.9)	13.8 (57.0-70.8)	5.0 (78.9-83.9)	12.0 (87.6-99.6)	/
	Knee[c]	5	3.82	8.0 (4.5-12.5)	11.1 (14.1-25.2)	5.0 (32.7-37.7)	7.4 (60.2-67.6)	5.1 (73.2-78.3)	/
	Ankle[c]	3	3.88	9.9 (0.6-10.5)	17.7 (13.9-31.6)	4.1 (62.6-66.6)			/

[a] $\alpha=0.017$; [b] $\alpha=0.008$; [c] $\alpha=0.006$; [d] $\alpha=0.004$

all $p \leq 0.001$ apart from [e] $p = 0.0035$

All relevant and significant suprathreshold clusters that were identified during the GC by the Hotteling's T^2 test for the vectors (i.e., components consisting of the combination of the individual motions per plane and type of waveform: sagittal kinematics, coronal kinematics, transversal kinematics, sagittal moments, powers) and the two-tailed t-tests for the segment or joint waveforms, are reported. The later are reported on the condition that (1) the Hotelling's T^2 test revealed significant differences on vector levels and (2) the results are clinically relevant, which was judged by the suprathreshold cluster's duration being longer or equal to 3% of the GC and the differences in the segment or joint waveforms between both gait patterns exceeding the respective SEM for the intra-rater intersession reliability in TD, reported by Kainz et al.[3], for 80% or more of the suprathreshold cluster's duration.

GC=gait cycle; n=number of identified suprathreshold clusters; SEM=standard error of measurement; T2*/t*=critical threshold needed to reject the null hypothesis; % extent (range)=extent of the identified suprathreshold cluster (start and end points of the suprathreshold cluster)

Table S9: Kinematic and kinetic differences between the flexion pattern (i.e., cluster three) and typically developing children.

		n	T2*/t*	Suprathreshold cluster 1 % extent (range)	Suprathreshold cluster 2 % extent (range)	Suprathreshold cluster 3 % extent (range)	Suprathreshold cluster 4 % extent (range)	Suprathreshold cluster 5 % extent (range)	Suprathreshold cluster 6 % extent (range)	Suprathreshold cluster 7 % extent (range)	SEM [3]
Sagittal kinematics	Vector[a]	1	18.89	100 (0-100)							
	Pelvis[d]	1	3.45	100 (0-100)							2.7
	Hip[d]	2	3.55	10.5 (0-10.5)	86.8 (13.2-100)						2.8
	Knee[d]	4	3.74	18.8 (5.6-24.4)	9.6 (36.7-46.2)	11.3 (54.8-66.1)	6.1 (93.9-100)				2.3
	Ankle[d]	3	3.81	3.5 (0-3.5)	35.5 (11.9-47.5)	39.7 (60.3-100)					2
Coronal kinematics	Vector[a]	2	13.88	10.5 (0-10.5)	78.1 (21.9-100)						
	Pelvis[b]	3	3.55	10.1 (0-10.1)	37.5 (21.9-59.4)	29.2 (70.8-100)					1.7
	Hip[b]	2	3.58	9.3 (28.6-37.9)[e]	28 (55-83)						2.5
Transversal kinematics	Vector[a]	2	16.76	92 (0-92)	8 (92-100)						
	Pelvis[c]	1	3.52	12.8 (0-12.8)							1.8
	Hip[c]	2	3.41	18.9 (6.2-25.1)	35.3 (56.7-92)						4.7
	Foot[c]	3	3.49	49.7 (0-49.7)	15.1 (65.7-80.8)	6.3 (93.7-100)					/
Sagittal Moments	Vector[a]	1	20.11	100 (0-100)							
	Hip[c]	3	4.05	32.2 (0-32.2)	27.9 (41.4-69.3)	6.5 (93.5-100)					0.05
	Knee[c]	5	3.87	3.6 (0-3.6)	16.1 (5.4-21.5)	20.8 (27.7-48.5)	21.1 (56.9-78)	9.4 (90.6-100)			0.05
	Ankle[c]	2	3.75	19.4 (0.3-19.7)	23.7 (31.0-54.7)						0.05
Coronal Moment	Hip[a]	2	3.55	6.4 (0-6.4)	11 (53.1-64.2)						0.05
Powers	Vector[a]	4	21.10	68.4 (0-68.4)	8.7 (71.9-80.6)	10.5 (87-97.5)	1.3 (98.7-100)				
	Hip[c]	3	4.07	15.2 (1.7-16.9)	22.3 (24-46.3)	18.3 (49.3-67.6)					/
	Knee[c]	7	4.31	3.4 (0-3.4)	8.5 (4.3-12.8)	12.3 (13.9-26.1)	9 (30.2-39.2)	18.8 (49.2-68)	4.6 (73.9-78.6)	8.8 (87.6-96.4)	/
	Ankle[c]	4	4.10	6.1 (2.5-8.5)	5 (13.5-18.5)	12 (43.4-55.3)	5.1 (62.6-67.7)				/

[a] $\alpha=0.017$; [b] $\alpha=0.008$; [c] $\alpha=0.006$; [d] $\alpha=0.004$

all $p \leq 0.001$ apart from [e] $p = 0.0013$

All relevant and significant suprathreshold clusters that were identified during the GC by the Hotteling's T^2 test for the vectors (i.e., components consisting of the combination of the individual motions per plane and type of waveform: sagittal kinematics, coronal kinematics, transversal kinematics, sagittal moments, powers) and the two-tailed t-tests for the segment or joint waveforms, are reported. The later are reported on the condition that (1) the Hotelling's T^2 test revealed significant differences on vector levels and (2) the results are clinically relevant, which was judged by the suprathreshold cluster's duration being longer or equal to 3% of the GC and the differences in the segment or joint waveforms between both gait patterns exceeding the respective SEM for the intra-rater intersession reliability in TD, reported by Kainz et al.[3], for 80% or more of the suprathreshold cluster's duration.

GC=gait cycle; n=number of identified suprathreshold clusters; SEM=standard error of measurement; T2*/t*=critical threshold needed to reject the null hypothesis; % extent (range)=extent of the identified suprathreshold cluster (start and end points of the suprathreshold cluster)

Table S10: Kinematic and kinetic vectors' comparisons among the three gait patterns in DMD

	n	X2*	Suprathreshold cluster 1<br>% extent (range)
Sagittal plane kinematics	1	23.75	100 (0-100)
Coronal plane kinematics	1	17.84	100 (0-100)
Transversal plane kinematics	1	21.04	100 (0-100)
Sagittal plane moments	1	21.21	100 (0-100)
Powers	1	22.05	100 (0-100)

All $p \leq 0.001$

DMD=Duchenne muscular dystrophy; MANOVA=multiple analysis of variance; n=number of identified suprathreshold clusters; X2*=critical threshold needed to reject the null hypothesis; % extent (range)=extent of the identified suprathreshold cluster (start and end points of the suprathreshold cluster)

Table S11: Kinematic and kinetic differences between the mildly affected gait pattern (i.e., cluster one) and the tiptoeing gait pattern (i.e., cluster two)

		n	T2*/t*	Suprathreshold cluster 1 % extent (range)	Suprathreshold cluster 2 % extent (range)	Suprathreshold cluster 3 % extent (range)	Suprathreshold cluster 4 % extent (range)	Suprathreshold cluster 5 % extent (range)	Suprathreshold cluster 6 % extent (range)	SEM [3,4]
Sagittal kinematics	Vector[a]	1	21.22	100 (0-100)						
	Trunk[d]	1	3.37	100 (0-100)						1.01
	Pelvis[d]	1	3.41	100 (0-100)						2.7
	Hip[d]	1	3.64	70.6 (28.6-99.2)						2.8
	Knee[d]	2	3.61	24.6 (1.8-26.3)	3.7 (45.7-49.4)					2.3
	Ankle[d]	1	3.85	100 (0-100)						2
Coronal kinematics	Vector[a]	1	16.21	100 (0-100)						
	Trunk[b]	3	3.49	32 (0-32)	25.8 (47.9-73.7)	8.4 (91.6-100)				0.83
	Pelvis[b]	3	3.59	22 (0-22)	37.6 (30.5-68.1)	24.3 (75.7-100)				1.7
	Hip[b]	2	3.58	63.1 (3.3-66.3)	16.7 (73.8-90.4)					2.5
Transversal kinematics	Vector[a]	1	18.89	100 (0-100)						
	Trunk[c]	3	3.52	37 (0-37)	37.5 (47.5-85.1)	4.1 (95.9-100)				1.11
	Pelvis[c]	2	3.76	42.2 (0-42.2)	39.4 (50.6-90)					1.8
	Hip[c]	3	3.61	9.1 (0-9.1)	53.4 (11.5-64.9)	9.6 (68.6-78.2)				4.7
	Foot[c]	3	3.58	4 (0-4)[e]	26 (13.1-39.1)	3.2 (96.8-100)				/
Sagittal Moments	Vector[a]	2	19.94	37.1 (0-37.1)	55.4 (41-96.4)					
	Hip[b]	4	3.83	11.7 (10.1-21.8)	3.1 (54.7-57.8)	9.9 (62.1-72.1)	19 (77-96)			0.05
	Knee[b]	1	3.81	33.1 (2.2-35.3)						0.05
	Ankle[b]	1	3.69	37.1 (0.1-37.1)						0.05
Coronal Moment	Hip[a]	4	3.56	6 (6.8-12.8)	3.4 (14.6-18)	17.8 (20.9-38.7)	8.8 (47.8-56.5)			0.05
Powers	Vector[a]	6	20.82	11.7 (0-11.7)	21 (12.7-33.7)	12.2 (44.6-56.7)	13.2 (58.6-71.8)	8.5 (75.4-83.9)	13.1 (86.9-100)	
	Hip[b]	4	3.84	5.2 (22.7-27.8)	9 (62.9-71.8)	6.5 (77.4-83.9)	13.1 (86.9-100)			/
	Knee[b]	4	4.22	4.4 (3.8-8.2)	11.8 (14.7-26.5)	10 (61.2-71.2)	3.3 (76.1-79.4)			/
	Ankle[b]	4	4.01	11 (0.6-11.6)	19.4 (14.3-33.7)	10.7 (45.4-56)	3.1 (58.6-61.7)			/

[a] α=0.017; [b] α=0.006; [c] α=0.004; [d] α=0.003; all p ≤ 0.001 apart from [e] p =0.0011

All relevant and significant suprathreshold clusters that were identified during the GC by the Hotteling's T^2 test for the vectors (i.e., components consisting of the combination of the individual motions per plane and type of waveform: sagittal kinematics, coronal kinematics, transversal kinematics, sagittal moments, powers) and the two-tailed t-test for the segment or joint waveforms, are reported. Suprathreshold clusters identified by the Hotteling's T^2 tests are reported on the condition that the MANOVA revealed significant differences on vector level. Suprathreshold clusters identified by the two-tailed t-tests are reported on the condition that (1) the MANOVA and Hotelling's T^2 test revealed significant differences on vector level and (2) the results are clinically relevant, which was judged by the suprathreshold cluster's duration being longer or equal to 3% of the GC and the differences in the segment or joint waveforms between both gait patterns exceeding the respective SEM for the intra-rater intersession reliability in TD, reported by Kainz et al.[3] (for pelvis and lower limb) and Wilken et al.[4] (for trunk), for 80% or more of the suprathreshold cluster's duration.

GC=gait cycle; n=number of identified suprathreshold clusters; SEM=standard error of measurement; T2*/t*=critical threshold needed to reject the null hypothesis; % extent (range)=extent of the identified suprathreshold cluster (start and end points of the suprathreshold cluster)

Table S12: Kinematic and kinetic differences between the tiptoeing gait pattern (i.e., cluster two) and the flexion pattern (i.e., cluster three)

		n	T2*/t*	Suprathreshold cluster 1 % extent (range)	Suprathreshold cluster 2 % extent (range)	Suprathreshold cluster 3 % extent (range)	Suprathreshold cluster 4 % extent (range)	Suprathreshold cluster 5 % extent (range)	Suprathreshold cluster 6 % extent (range)	Suprathreshold cluster 7 % extent (range)	SEM [3,4]
Sagittal kinematics	Vector[a]	1	21.47	100 (0-100)							
	Trunk[d]	3	3.43	9.8 (0-9.8)	35.8 (20.2-56)	20.8 (79.2-100)					1.01
	Pelvis[d]	2	3.40	28.5 (0-28.5)	34.2 (44.4-78.6)						2.7
	Hip[d]	1	3.62	61.8 (0-61.8)							2.8
	Knee[d]	4	3.71	42.1 (0-42.1)	5 (57.3-62.3)	13.3 (77-90.3)	4.1 (95.9-100)				2.3
	Ankle[d]	1	3.37	100 (0-100)							2
Coronal kinematics	Vector[a]	2	16.77	51.7 (0-51.7)	39 (61-100)						
	Trunk[b]	2	3.52	22.6 (0-22.6)	10.1 (89.9-100)						0.83
	Pelvis[b]	2	3.52	29.9 (4.6-34.5)	14.7 (61-75.8)						1.7
	Hip[b]	1	3.46	16.1 (73.1-89.2)							2.5
Transversal kinematics	Vector[a]	2	19.58	88.6 (0-88.6)	6.8 (93.2-100)[e]						
	Trunk[c]	2	3.64	39.2 (0-39.2)	39.7 (48.8-88.6)						1.11
	Pelvis[c]	2	3.53	40.9 (1-42)	35.4 (53.1-88.6)						1.8
	Hip[c]	1	3.90	8.8 (70.8-79.7)							4.7
	Foot[c]	2	3.72	4.5 (69.6-74.2)	6.8 (93.2-100)						/
Sagittal Moments	Vector[a]	4	19.90	53.5 (0-53.5)	9.7 (61.6-71.3)	12.6 (78.1-90.7)	6.6 (93.4-100)				
	Hip[b]	4	3.90	10.8 (42.8-53.5)	7.7 (63-70.8)	8.2 (79.7-87.9)	3.7 (96.3-100)				0.05
	Knee[b]	2	3.85	43.7 (0.0-43.7)	6.2 (93.8-100)						0.05
	Ankle[b]	1	3.92	19.7 (4.3-24.0)							0.05
Coronal Moment	Hip[a]	4	3.53	5.9 (6.7-12.6)	4 (14.2-18.3)	21.4 (21.8-43.2)	11.6 (57.1-68.8)				0.05
Powers	Vector[a]	6	20.80	34.8 (0-34.8)	26.8 (36.7-63.6)	5.3 (65.1-70.4)	1.6 (74.2-75.9)[f]	3.9 (80.4-84.3)	13.1 (86.9-100)		/
	Hip[b]	7	4.16	3.4 (0.0-3.4)	6.9 (11.3-18.2)	4.9 (24.8-29.8)	10 (37.2-47.2)	9.4 (50.8-60.2)	4.6 (65.5-70.2)	12.6 (87.4-100)	/
	Knee[b]	2	3.94	6.7 (47.6-54.3)	10.1 (89.9-100)						/
	Ankle[b]	3	4.15	10.9 (0.5-11.4)	20.6 (14.2-34.8)	13.4 (44.2-57.5)					/

[a] $\alpha=0.017$; [b] $\alpha=0.006$; [c] $\alpha=0.004$; [d] $\alpha=0.003$; all $p \leq 0.001$ apart from [e] $p = 0.0011$ and [f] $p=0.0053$

All relevant and significant suprathreshold clusters that were identified during the GC by the Hotteling's T^2 test for the vectors (i.e., components consisting of the combination of the individual motions per plane and type of waveform: sagittal kinematics, coronal kinematics, transversal kinematics, sagittal moments, powers) and the two-tailed t-test for the segment or joint waveforms, are reported. Suprathreshold clusters identified by the Hotteling's T^2 tests are reported on the condition that the MANOVA revealed significant differences on vector level. Suprathreshold clusters identified by the two-tailed t-tests are reported on the condition that (1) the MANOVA and Hotelling's T^2 test revealed significant differences on vector level and (2) the results are clinically relevant, which was judged by the suprathreshold cluster's duration being longer or equal to 3% of the GC and the differences in the segment or joint waveforms between both gait patterns exceeding the respective SEM for the intra-rater intersession reliability in TD, reported by Kainz et al.[3] (for pelvis and lower limb) and Wilken et al.[4] (for trunk), for 80% or more of the suprathreshold cluster's duration.

GC=gait cycle; n=number of identified suprathreshold clusters; SEM=standard error of measurement; T2*/t*=critical threshold needed to reject the null hypothesis; % extent (range)=extent of the identified suprathreshold cluster (start and end points of the suprathreshold cluster)

Table S13: Kinematic and kinetic differences between the mildly affected gait pattern (i.e., cluster one) and the flexion gait pattern (i.e., cluster three)

		n	T2*/t*	Suprathreshold cluster 1 % extent (range)	Suprathreshold cluster 2 % extent (range)	Suprathreshold cluster 3 % extent (range)	Suprathreshold cluster 4 % extent (range)	Suprathreshold cluster 5 % extent (range)	SEM [3,4]
Sagittal kinematics	Vector[a]	1	18.98	100 (0-100)					
	Trunk[d]	1	3.17	100 (0-100)					1.01
	Pelvis[d]	1	3.42	100 (0-100)					2.70
	Hip[d]	1	3.51	100 (0-100)					2.80
	Knee[d]	3	3.72	33.6 (29.2-62.7)	18.5 (72.3-90.8)	4.7 (95.3-100)			2.30
	Ankle[d]	1	3.87	86.3 (13.7-100)					2.00
Coronal kinematics	Vector[a]	1	15.01	100 (0-100)					
	Trunk[b]	2	3.37	24.1 (0.7-24.9)	19.9 (54.3-74.1)				0.83
	Pelvis[b]	3	3.34	7.3 (0-7.3)	45.7 (13.6-59.3)	34.7 (65.3-100)			1.70
	Hip[b]	1	3.45	61.8 (5.2-67)					2.50
Transversal kinematics	Vector[a]	2	17.67	68.8 (0-68.8)	28.2 (71.8-100)				
	Trunk[c]	1	3.42	16.2 (78.4-94.6)					1.11
	Pelvis[c]	0	3.64						1.80
	Hip[c]	2	3.60	5 (0-5)	54.9 (13.6-68.5)				4.70
	Foot[c]	2	3.45	65.2 (0-65.2)	10.5 (89.5-100)				/
Sagittal Moments	Vector[a]	1	18.23	100 (0-100)					
	Hip[b]	3	3.79	5.3 (0-5.3)	9.2 (12.5-21.7)	19.7 (40.8-60.5)			0.05
	Knee[b]	2	3.75	17.2 (4-21.2)	15.2 (34.5-49.7)				0.05
	Ankle[b]	1	3.63	53.2 (0.3-53.5)					0.05
Coronal Moment	Hip[a]	1	3.47	30.1 (39.8-70)					0.05
Powers	Vector[a]	4	18.23	29.4 (0-29.4)	10.9 (35.7-46.7)	36.7 (49.9-86.6)	10.7 (89.3-100)		
	Hip[b]	4	3.86	4.1 (0-4.1)	7.9 (17.0-24.9)	10.6 (36.1-46.7)	32 (50.8-82.8)		/
	Knee[b]	5	3.81	6.2 (3.3-9.4)	16.3 (12.9-29.2)	23.5 (49.9-73.4)	8.9 (77.8-86.6)	8.8 (89.3-98.2)	/
	Ankle[b]	3	3.91	6.4 (2.4-8.8)	5.8 (56.0-61.8)	4.6 (62.8-67.4)			/

[a] $\alpha=0.017$; [b] $\alpha=0.006$; [c] $\alpha=0.004$; [d] $\alpha=0.003$; all $p \leq 0.001$

All relevant and significant suprathreshold clusters that were identified during the GC by the Hotteling's T^2 test for the vectors (i.e., components consisting of the combination of the individual motions per plane and type of waveform: sagittal kinematics, coronal kinematics, transversal kinematics, sagittal moments, powers) and the two-tailed t-test for the segment or joint waveforms, are reported. Suprathreshold clusters identified by the Hotteling's T^2 tests are reported on the condition that the MANOVA revealed significant differences on vector level. Suprathreshold clusters identified by the two-tailed t-tests are reported on the condition that (1) the MANOVA and Hotelling's T^2 test revealed significant differences on vector level and (2) the results are clinically relevant, which was judged by the suprathreshold cluster's duration being longer or equal to 3% of the GC and the differences in the segment or joint waveforms between both gait patterns exceeding the respective SEM for the intra-rater intersession reliability in TD, reported by Kainz et al.[3] (for pelvis and lower limb) and Wilken et al.[4] (for trunk), for 80% or more of the suprathreshold cluster's duration.

GC=gait cycle; n=number of identified suprathreshold clusters; SEM=standard error of measurement; T2*/t*=critical threshold needed to reject the null hypothesis; % extent (range)=extent of the identified suprathreshold cluster (start and end points of the suprathreshold cluster)

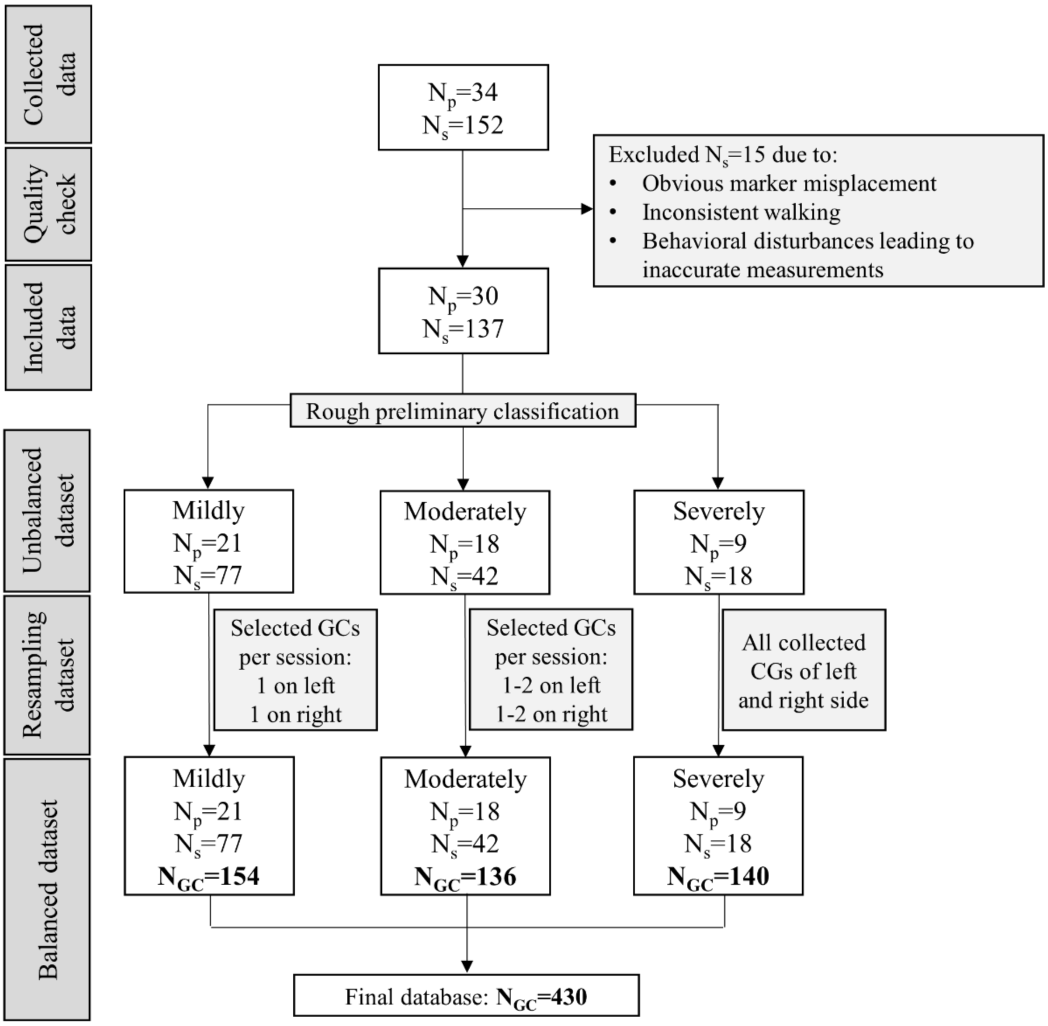

Figure S1: An overview of the collected data, the included data and the resampling process through a rough preliminary classification based on two previous classifications and insights from the clinical experts at Clinical Motion Analysis Laboratory in the University Hospital Leuven to achieve a balanced dataset.

Abbreviations in alphabetic order: GCs=gait cycles; N_{GC}=number of gait cycles; N_p=number of participants; N_s=number of 3D gait sessions

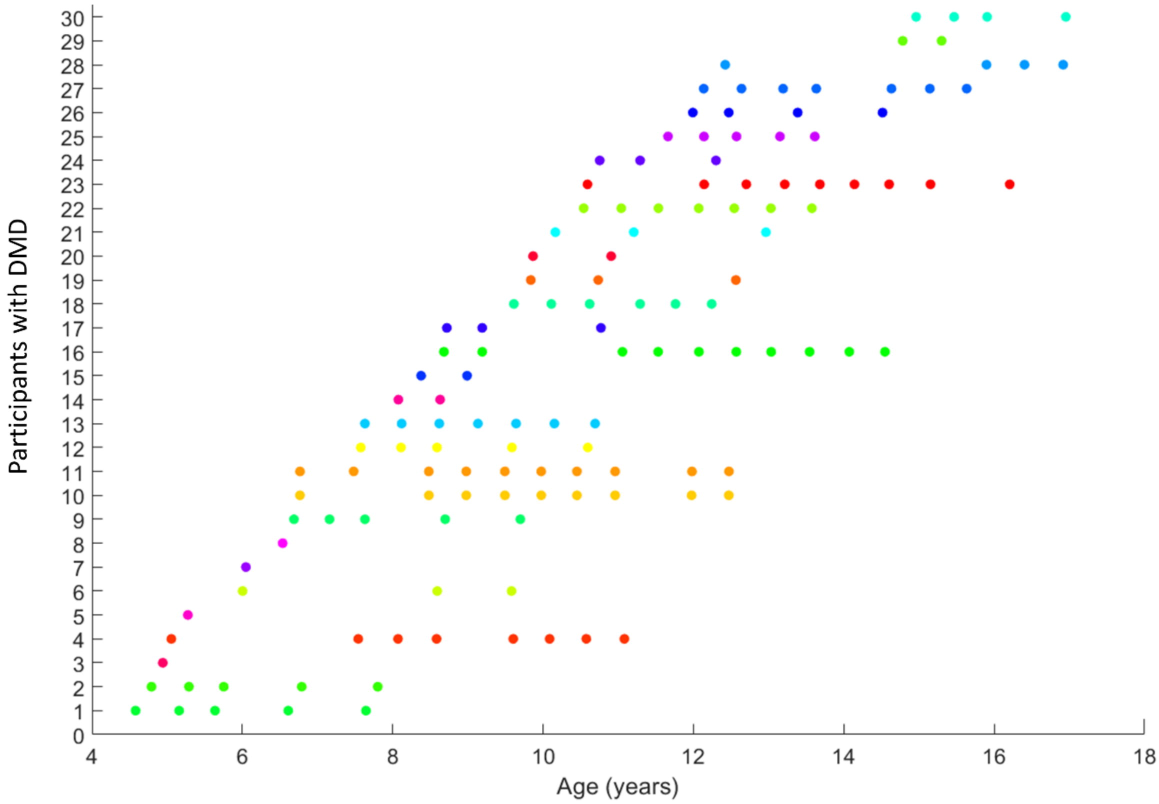

Figure S2: Mixed cross-sectional longitudinal dataset. Overview of the ages at the included measurements for the boys with DMD. Each color represents one patient.
Abbreviations in alphabetic order: DMD=Duchenne muscular dystrophy

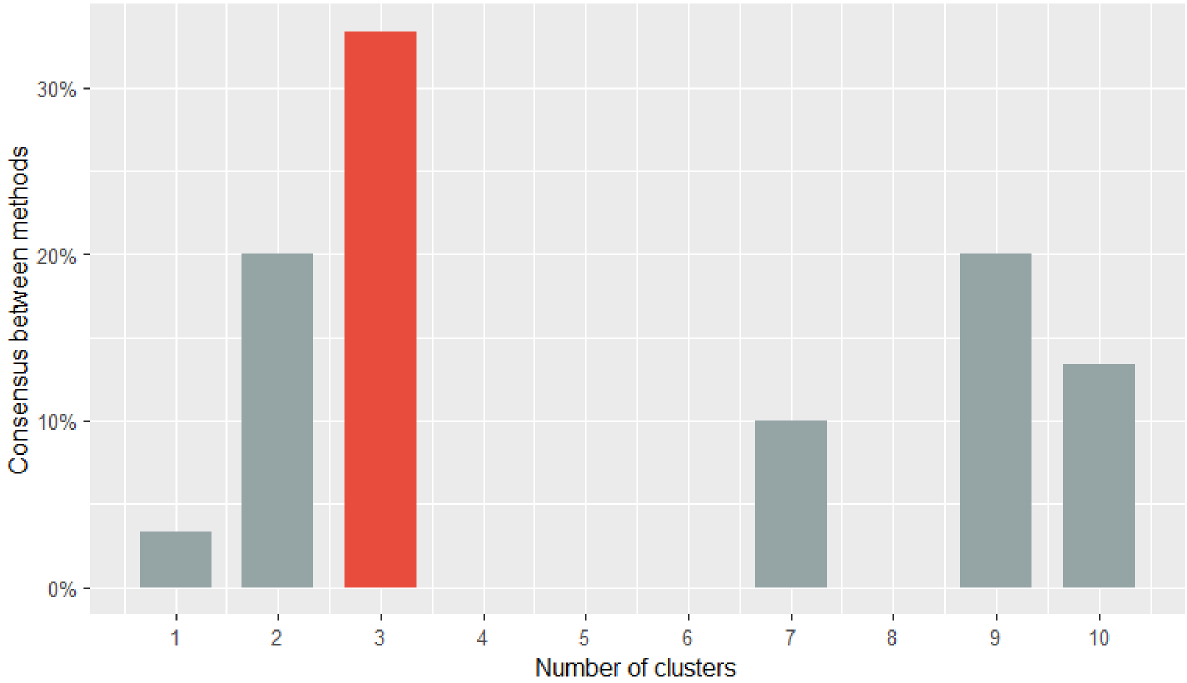

Figure S3: The percentage of consensus on the optimal number of clusters between 30 methods for one- to ten-cluster configuration[5]. The choice of three clusters is supported by 10 (33.3%) out of the 30 methods (Marriot, trcovw, Tracew, Friedman, Duda, Pseudot2, Beale, Ratkowsky, Ball, PtBiserial). One method indicated one cluster (3.3%), six methods indicated two clusters (20%), three methods indicated seven clusters (10%), six methods indicated nine clusters (20%) and four methods indicated ten clusters (13%), as the optimal number of clusters.

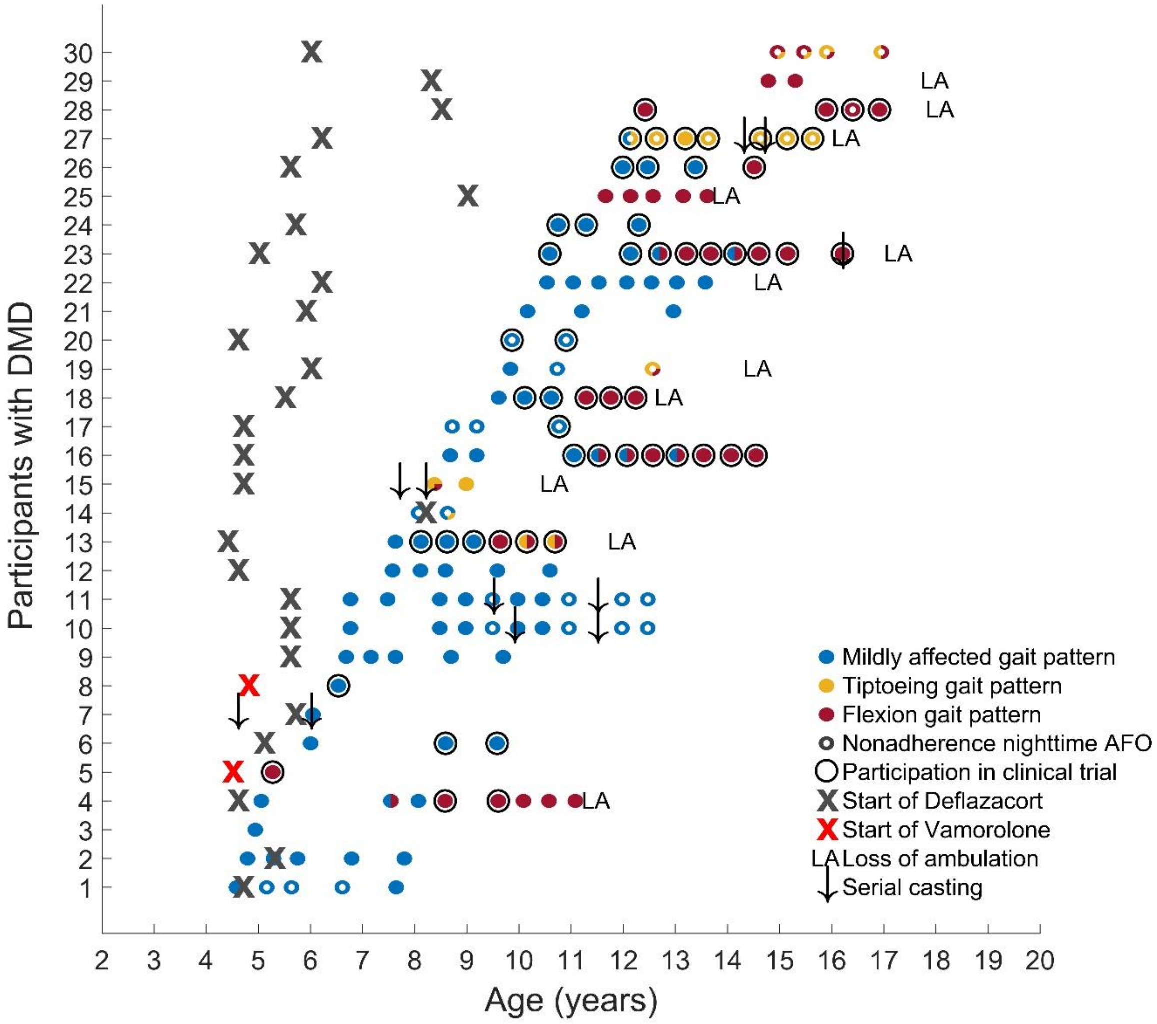

Figure S4: Overview of the assignment to the gait patterns of the participants' repeated 3DGA-sessions and the participants' <u>medical and clinical background,</u> along with indications of overlap between patterns. Overlap between gait patterns for a particular 3DGA-session is displayed by coloring the marker ¾ or ½, depending on the proportion of the number of GCs assigned to a particular gait pattern. In case of ¾ division, the classification of the session was interpreted according to the majority of the GCs' assignment. The ½ division was always related to asymmetry. These sessions with overlap could also indicate an "intermediate" stage between gait patterns. Patients 4, 16, 18, 23 and 26, with good adherence to nighttime AFO, almost no serial casting (except once for patient 23) and consistent participation in clinical trials with disease-modifying medication (patients 16, 18, 23 and 26), transitioned from the mildly affected to the flexion pattern. Patients 4, 16 and 23 showed a period where they were assigned to both the mildly affected and flexion gait pattern, which could indicate an "intermediate" stage. Patients 19 and 27, with nonadherence to nighttime AFO in both and repeated serial casting as well as clinical trial participation with disease-modifying medication for patient 27, transitioned from the mildly affected to the tiptoeing gait pattern. Patient 13, with good adherence to nighttime AFO and clinical trial participation with disease-modifying medication, first showed a transition from the mildly affected to the flexion gait pattern and then demonstrated both the tiptoeing and the flexion gait pattern. Patient 30, with nonadherence to nighttime AFO, first transitioned from the flexion to the tiptoeing gait pattern and then demonstrated both the tiptoeing and flexion gait pattern. However, overlap was detected for all of his sessions, which could indicate an "intermediate" stage between these two gait patterns.

Abbreviations in alphabetic order: AFO=ankle foot orthosis; DMD=Duchenne muscular dystrophy; GC=gait cycle; LA=loss of ambulation

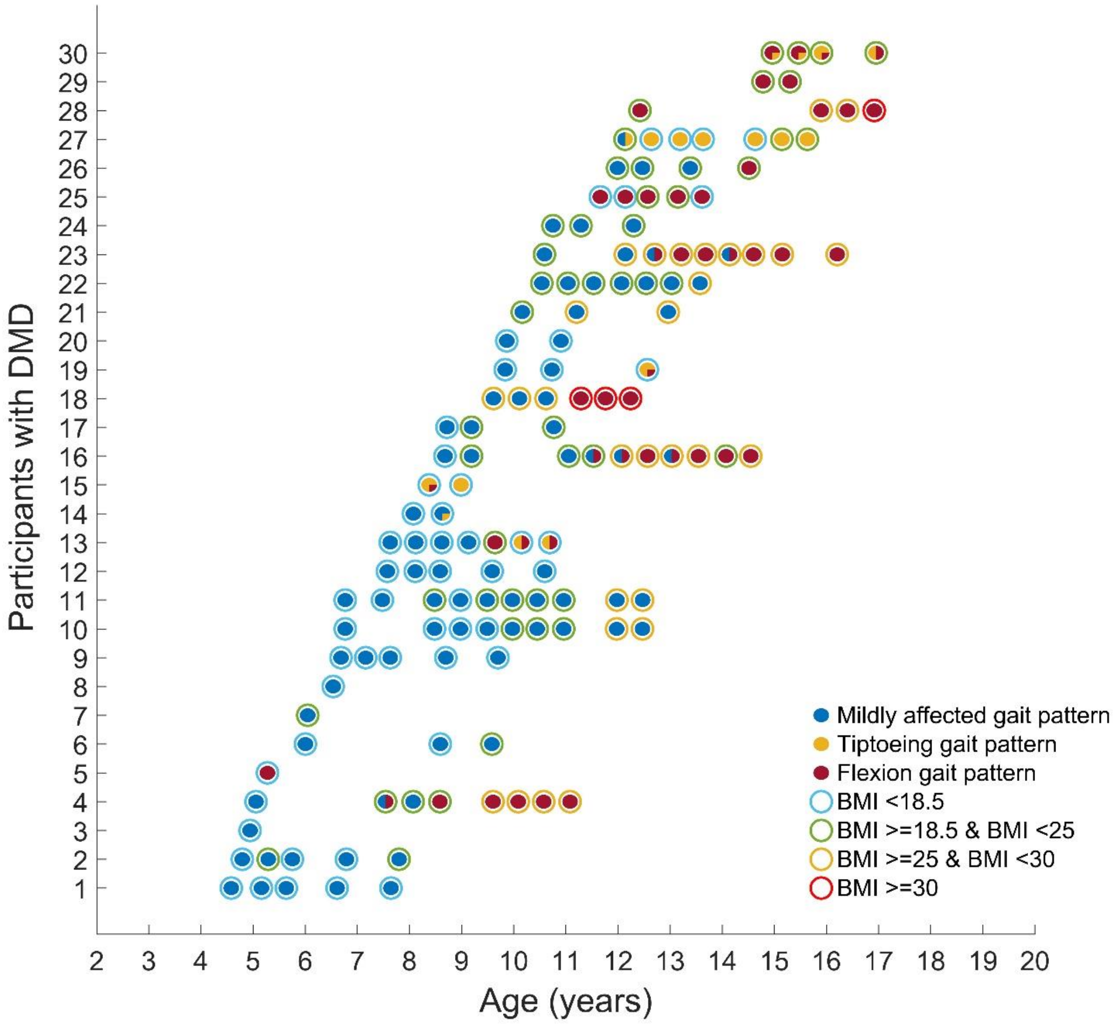

Figure S5: Overview of the assignment to the gait patterns of the participants' repeated 3DGA-sessions and the participants' BMI category, along with indications of overlap between patterns. Overlap between gait patterns for a particular 3DGA-session is displayed by coloring the marker ¾ or ½, depending on the proportion of the number of GCs assigned to a particular gait pattern. In case of ¾ division, the classification of the session was interpreted according to the majority of the GCs' assignment. The ½ division was always related to asymmetry. These sessions with overlap could also indicate an "intermediate" stage between gait patterns. The transition from the mildly affected to the flexion gait pattern appear to occur along with an increasing BMI in patients 4, 16, 18 and 23.

Abbreviations in alphabetic order: BMI=body mass index; DMD=Duchenne muscular dystrophy; GC=gait cycle

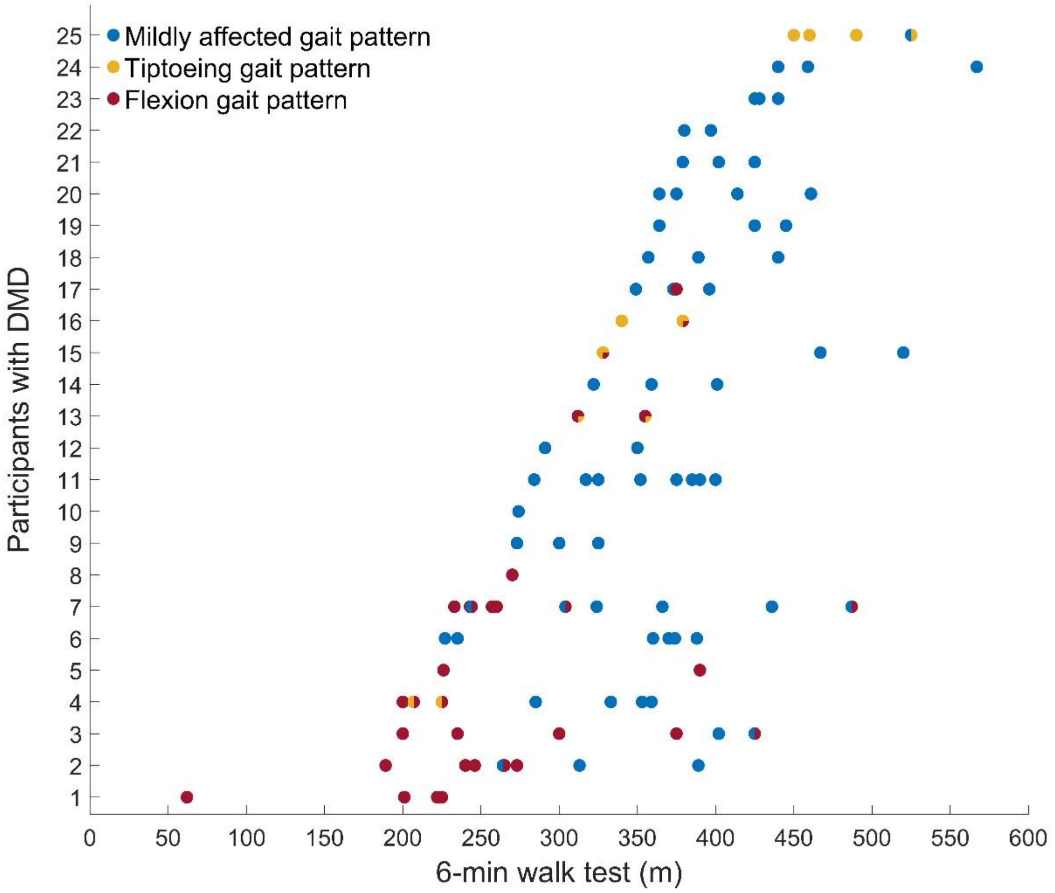

Figure S6: Visual exploration of the <u>occurrence of gait patterns along with the scores on the 6-min walk test.</u> The flexion gait pattern appears to occur along with shorter distances on the 6-min walk test, while the other two gait patterns appear to occur along with longer distances on the 6-min walk test. Overlap between gait patterns for a particular 3DGA-session is displayed by coloring the marker ¾ or ½, depending on the proportion of the number of GCs assigned to a particular gait pattern.

Abbreviations in alphabetic order: DMD=Duchenne muscular dystrophy; GC=gait cycle; m=meter; min=minutes

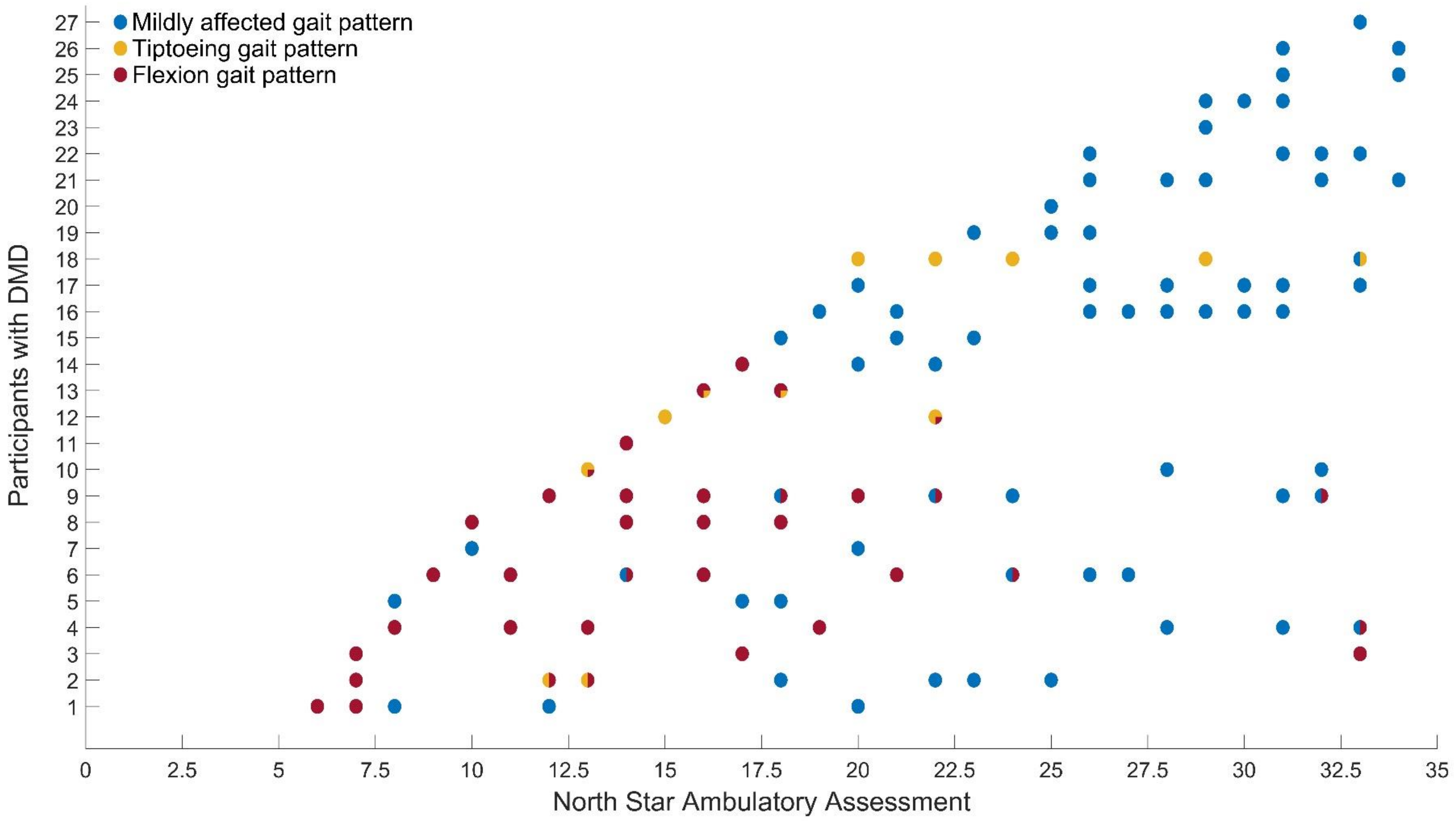

Figure S7: Visual exploration of the occurrence of gait patterns along with the scores on the North Star Ambulatory Assessment. The flexion gait pattern appears to occur along with lower values on the North Star Ambulatory Assessment, while the mildly affected gait pattern appears to occur along with higher values on the North Star Ambulatory Assessment. Overlap between gait patterns for a particular 3DGA-session is displayed by coloring the marker ¾ or ½, depending on the proportion of the number of GCs assigned to a particular gait pattern.
Abbreviations in alphabetic order: DMD=Duchenne muscular dystrophy; GC=gait cycle

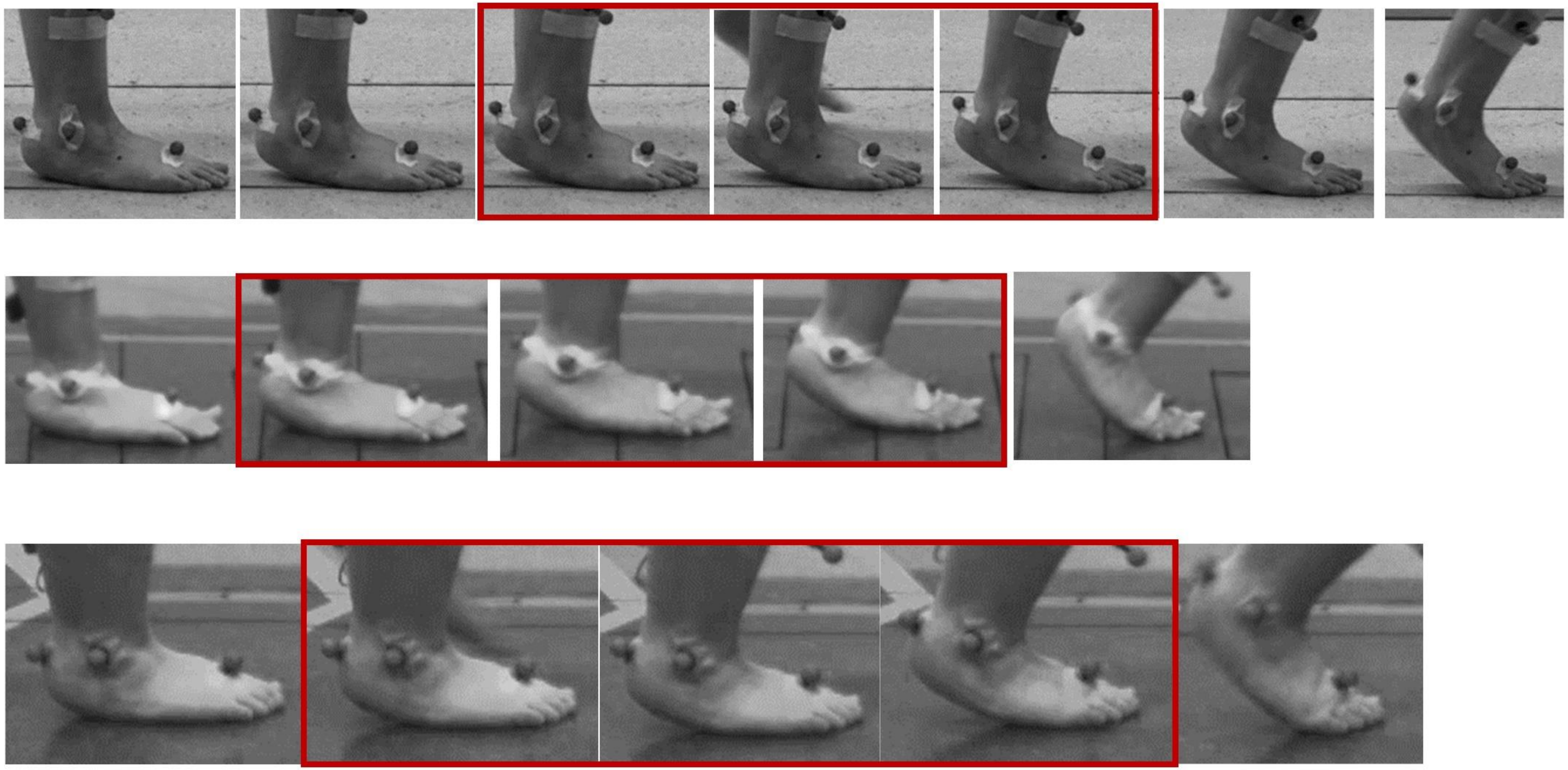

Figure S8: Three examples of boys with DMD who showed a midfoot break during the weightbearing phase of gait. The red rectangles highlight the pictures where the midfoot break is clearly present. A midfoot break is defined as an abnormally increased motion through the midfoot. Due to a gastrocnemius contracture, the forward progression of the tibia is impeded[6]. If the midfoot is also flexible, the motion to ensure forward progression will be transferred from the ankle to the midfoot/tarsal joint[6], resulting in an abnormally increased motion through the midfoot (i.e., midfoot break). More flexible feet with larger arch drops and lower arch rigidity when bearing weight have been previously reported in pediatric obesity[7].

References

1. Sutherland, D. H. *et al.* The pathomechanics of gait in Duchenne Muscular Dystrophy. *Dev. Med. Child Neurol.* **23**, 3–22 (1981).
2. Sidak, Z. Rectangular Confidence Regions for the Means of Multivariate Normal Distributions. *J. Am. Stat. Assoc.* **62**, 626–633 (1967).
3. Kainz, H. *et al.* Reliability of four models for clinical gait analysis. *Gait Posture* **54**, 325–331 (2017).
4. Wilken, J. M., Rodriguez, K. M., Brawner, M. & Darter, B. J. Reliability and minimal detectible change values for gait kinematics and kinetics in healthy adults. *Gait Posture* **35**, 301–307 (2012).
5. Charrad, M., Ghazzali, N., Boiteau, V. & Niknafs, A. Nbclust: An R package for determining the relevant number of clusters in a data set. *J. Stat. Softw.* **61**, 1–36 (2014).
6. Amis, J. The Split Second Effect: The Mechanism of How Equinus Can Damage the Human Foot and Ankle. *Front. Surg.* **3**, 1–10 (2016).
7. Shultz, S. P., Sitler, M. R., Tierney, R. T., Hillstrom, H. J. & Song, J. Consequences of pediatric obesity on the foot and ankle complex. *J. Am. Podiatr. Med. Assoc.* **102**, 5–12 (2012).
